# Pourbaix-Guided Mineralization and Site-Selective Photoluminescence Properties of Rare Earth Substituted B-Type Carbonated Hydroxyapatite Nanocrystals

**DOI:** 10.3390/molecules26030540

**Published:** 2021-01-21

**Authors:** Peng Liu, Zhengqiang Li, Long Yuan, Xiaolin Sun, Yanmin Zhou

**Affiliations:** 1 School of Stomatology, Jilin University, Changchun 130021, China; pengliu18@mails.jlu.edu.cn (P.L.); zqli13@mails.jlu.edu.cn (Z.L.); sunxiaolin@jlu.edu.cn (X.S.); 2 Key Laboratory of Functional Materials Physics and Chemistry of the Ministry of Education, College of Physics, Jilin Normal University, Changchun 130103, China

**Keywords:** carbonated hydroxyapatite, hydrothermal method, rare-earth labeling, site-selective occupancy

## Abstract

Rare-earth labeling in biological apatite could provide critical information for the pathologic transition (osteoclastic) and physiologic regeneration (osteogenesis) of bone and teeth because of their characteristic site-sensitive fluorescence in different coordinative conditions of various tissues in many biological processes. However, the rare-earth labeling method for biological apatites, i.e., carbonated-hydroxyapatite, has been rarely found in the literature. In this paper, we report a Pourbaix-diagram guided mineralizing strategy to controllable carbonation and doping of rare-earth ions in the hydroxyapatite (HA) lattice. The carbonation process of hydroxyapatite was achieved by controllable mineralization in hydrothermal condition with K_2_CO_3_ as the carbonate source, which results into the pure B-type carbonated hydroxyapatite (CHA) with tunable carbonate substitution degree. All of the as-synthesized materials crystalized into P63/m (No. 176) space group with the lattice parameter of a decreases and c increases with the increasing of carbonate content in the reactants. Structural refinement results revealed that the substitution of planar CO_3_^2−^ is superimposed on one of the faces of PO_4_^3−^ tetrahedral sub-units with a rotation angle of 30° in reference to c-axis. All of the hydrothermally synthesized CHA nanocrystals show hexagonal rod-like morphology with the length of 70–110 nm and diameter of 21–35 nm, and the decreasing length/diameter ratio from 3.61 to 2.96 from low to high carbonated level of the samples. Five rare-earth cations, of Pr^3+^, Sm^3+^, Eu^3+^, Tb^3+^, and Ho^3+^, were used as possible probe ions that can be doped into either HA or CHA lattice. The site-preference of Tb^3+^ doping is the same in the crystallographic site of HA and CHA according to characteristic emission peaks of ^5^D_4_–^7^F*_j_* (*j* = 3–6) transitions in their photoluminescent spectroscopy. Our work provides a controllable carbonation method for rare-earth labeling hydroxyapatite nanomaterials with potential biologically active implant powders for bone repair and tissue regeneration.

## 1. Introduction

Bone and teeth are the fundamental inorganic infrastructure for most animals as well as humankind, which form in a continuous biologically-controlled mineralization process for hydroxyapatite crystallization with hierarchy structures [1]. The hierarchical organization of bone tissues begin at the formation and assemble of hydroxyapatite at nanoscale that template by collagen fibril and encoded by various proteins [2], which could be delicately controlled by various enzymes in the biological mineralization process [3]. Although the most important inorganic component in hard tissues of animal and human is generally called as hydroxyapatite (HA), they are generally composed of various ions’ substituted forms with varied contents of carbonate for different bones, rather than pure phase hydroxyapatite with the nominal composition of Ca_10_(PO_4_)_6_(OH)_2_. As the main constituent of human bones, calcium orthophosphates form as poorly crystalline state with non-stoichiometric of Ca-deficient and carbonated HA, usually regarded as biological apatite. The carbonate composition varies greatly from different tissues, such as in enamel (3.5 wt%), dentin (5.6 wt%), and bone (7.4 wt%) [4], which could be ascribed to the different mineralization processes either at the periosteal (outer) surface or embedded in an organic matrix biomineralized with the assistance of collagen [5,6]. The doping of carbonate and other ions could tune the physiologic mineralizing process and mechanical performance in different tissues in the body. For example, the carbonate content in the crystal lattice determines the elastic properties of biological apatite [7]. The pure hydroxyapatite never founds in any biological systems, with all of the known apatite phase in biology composed of Ca-deficient, cation-substitution for Ca and anions of carbonate or fluoride for PO_4_^3−^ and OH^−^, respectively. Although as much as 72 elements could be doped into hydroxyapatite lattice with the possibility to tune its biological performance, nearly all of the calcium phosphates cements could not to exceed natural bone in compressive and tensile strengths [8]. Recently, Sato et al. demonstrated that the carbonated hydroxyapatite with a commercial name of Cytrans showed superior bone healing ability for vertical bone defect than that of β-tricalcium phosphate, hydroxyapatite, and bovine-derived heterogeneous bone grafting materials [9]. However, almost all of the known bone implanted powder materials were either obtained from natural bones by a series complex treatment procedure and the calcination in high temperature processes with expensive price or the synthesized pure hydroxyapatites with long bone regeneration period. Therefore, it is important to find an effective method to synthesize suitable alternative of biological apatite materials that could meet the requirement of bone substitution with highly efficient implantation.

Hydroxyapatite powder has long been recognized as the most promising bone implant materials because of its merits of similarity in structure with natural bone, easy to prepare, low-cost, etc. For example, nanostructures of hydroxyapatite have been reported to show excellent mechanical and biocompatible properties with potential biomedical applications in artificial bone and teeth, antibacterial and anticancer drug carrier, and cellular information transporters [10]. Recently, epitaxy growth of hydroxyapatite from the precursor of small calcium phosphate ionic clusters has been demonstrated as an effective route to repair tooth enamel due to the high reactivity of the calcium phosphate clusters [11]. However, the natural bones are grown as highly hierarchical structure with chemical gradients to adapt the residual stresses for the mechanical support at different positions [12], which implies that the fixed component could not meet the needs for bone formation and regeneration via only pure phase hydroxyapatite supply, which exhibits good osteoconductivity and regeneration of bone [13]. Carbonated hydroxyapatite would be a better alternative with two feasible substitution possibilities, which generates three structural types: (i) A-type, by substitution of OH^−^ at *c*-axis in the form of Ca_10_(PO_4_)_6_(OH)_2−2x_(CO_3_)_x_ with the x-value up to 1 for the fully-substituted Ca_10_(PO_4_)_6_(CO_3_) [14]; (ii) B-type, by substitution of PO_4_^3−^ ion in the form of Ca_10−x_Na_x_(PO_4_)_6−x_(CO_3_)_x_(OH)_2_; (iii) AB-type, with the various composition of the CO_3_^2−^ substitution both at the PO_4_^3−^ and OH^−^ site simultaneously [15]. The biological apatite is B-type hydroxyapatite that contains 4–6% of carbonate by weight, which shows composition dependent functions for different bone tissues and even in the same bone tissue, the composition still varies at different sites [16]. Therefore, carbonated hydroxyapatites with well controlled composition would be a superior bone implant powders than pure hydroxyapatite of Ca_10_(PO_4_)_6_(OH)_2_ and the complex composition of implant powders of commercial products.

Many experimental and theoretical works have been reported to synthesize carbonated hydroxyapatite materials and analyse the thermodynamic stability of the already known three types. A-type carbonated hydroxyapatite was obtained by sintering the pure HA in the atmosphere of CO_2_ at the temperature over 900 °C for at least 15 h [17,18], with its carbonate anions tuneable mobility and exchangeability in the channels [19]. Nordström et al. developed a socking method that treated HA in saturated carbon dioxide solution for up to two months to reach a suitable ionic exchanged carbonated ratio in the hydroxyapatite lattice [20]. Apart from these methods, no other routes have been reported to synthesize A-type CHA feasibly. The charge of A-type CHA is compensated by formation of OH vacancies, while B-type material by compensation of the combination of Ca^2+^ and OH^−^ deficiencies. The formation of B-type with a compensation of Ca^2+^ vacancy and hydrogen bonded phosphate is energy-favoured according to the first principle prediction [21]. However, B-type CHA is not suitable to be synthesized via thermal treatment method due to the various phases’ formation with the loss of hydroxide and carbonate composition up to the high Ca/P phase of tri-calcium phosphate (β-TCP) [22]. Hydroxyapatite phase is prone to be stable at high pH, in which the reactant solvent system provides enough mineralizer concentration to reach a better Ca/P ratio and higher carbonate content for the products [23]. The mineralizer contains Na^+^ is prone to results in the Na-bearing B-type or AB-type CHA phases with the charge compensation from CO_3_^2−^ substitution of PO_4_^3−^ [24]. The defects in B-type CHA resulted in acid and basic surface site that could promote catalytic performance of ethanol to hydrocarbon conversion [25]. However, the controllable carbonation method of B-type CHA with different carbonate contents is still not found in literature.

Rare-earth doped hydroxyapatites have been utilized to implant into bone tissue as a biomarker, such as monitoring the delivery of proteins [26] and tracking the implanted scaffold during bone regeneration [27]. Due to the similarity in the ionic size and coordinative requirements, lanthanides are good substitutes for Ca^2+^ site in calcium-based phosphate, especially for hydroxyapatite [28]. Rare-earth element Y and Sm doping in hydroxyapatite has been found a promotion effect for the osteoblast adhesion to improve the performance of orthopaedic implants [29,30]. However, most of rare-earth doped hydroxyapatite literatures were focused on the preparation and effect of Eu-doping. Eu^3+^ and Gd^3+^ co-doped hydroxyapatite have been synthesized via a microwave-assistant method, which shows a multifunction performance for both photoluminescenct and magnetic applications for better biomedical imaging [31]. Other rare-earth elements, such as Yb^3+^, Tm^3+^, have also been reported to dope in hydroxyapatites with up- and down-conversion performance for potential multimodal fluorescent imaging [32]. Crystallographic site selective occupancy of rare-earth ions in phosphate is possible for all sites that Ca^2+^ occupied, for example in Ca_10_M(PO_4_)_7_, Eu^2+^ could doping at any site of Ca(1), Ca(2), Ca(3), Na(4), and Ca(5), except the last Ca(5) site [33]. The selective occupancy of rare-earth in calcium phosphates could provide exact photoluminescence marker for monitoring the biomineralization process. Li et al. suggested that terbium (Tb) could be doped into the crystal lattice of hydroxyapatite nanocrystals as the biomarker for traceable fluorescence of intracellular interactions [34]; however, the in vivo experiment demonstrate that the HA implanted into the bone tissue still treated as foreign materials with long degradation period in bone repairing process [35]. Meanwhile, the pure hydroxyapatite and their rare-earth labelled compounds are still applied as bone repair and regeneration materials [36], which increased the repair period and increase the risk of implant failure. New materials with high performance for bone repair and tissue regeneration are still in need. Recently, Zn has also been doped in hydroxyapatite as a fingerprint for the osteogenic differentiation demonstrated the biomineralization in the extracellular matrix [37]. In previous work, we found the mineralizer induced crystal morphology evolution and site-selective occupancy of Eu^3+^ doped Ca_10_(PO_4_)_6_(OH)_2_ nanorods in hydrothermal condition [38]. However, the pure hydroxyapatite obtained by hydrothermal method was shown as high crystallinity and difficult to decompose in vivo as bone filling materials [39]. Although carbonated hydroxyapatite has been proved to be as superior implant materials with the biological properties approaching that of commercially available bovine xenograft (Bio-Oss) [40], most works still concentrated on the RE-labelling for pure phase HA [41], rather than biological active apatite (CHA). In this paper, we focus on the controllable carbonation of hydroxyapatite of B-type based on the themodynamic data in Pourbaix diagram system. Then, the possibility of rare-earth (RE = Pr, Sm, Eu, Tb, Ho) doping in the crystal lattice of hydroxyapatite and carbonated hydroxyapatite and their site preference were considered. In consideration of the ionic size of K^+^ (1.38 Å) is larger than that of Ca^2+^ (1.00 Å) and Na^+^ (1.02 Å), while that of Na^+^ and Ca^2+^ are nearly the same for the six-coordinated state, the occupancy of Na^+^ in Ca^2+^ lattice is energetically favored and therefore the substitution of Ca^2+^ by Na^+^ is inevitable [15]. To exclude the effect of Na^+^ inclusion on the structure and luminescent biomarker properties, K_2_CO_3_ was used as the sole carbonate source in this paper.

## 2. Results and Discussion

### 2.1. Pourbaix Diagram of the Chemical Balance in the Calcium, Phosphates, Cabonates in Aqueous System

To resolve the crystallization mechanism of HA and CHA in aqueous system, the thermodynamic stable region of each species of calcium, phosphate, and carbonate should be taken into a general consideration in various pH and temperature regions. Phosphoric acid is a triprotic acid with the pK_a_(1) = 2.21, pK_a_(2) = 7.21, pK_a_(3) = 12.67 at room temperature. At the human body temperature (37 °C), phosphate could possibly exist as four states with the pH range from 1 to 14 (Figure S1), i.e., H_3_PO_4_ (pH < 2.1), H_2_PO_4_^−^ (2.1 < pH < 7.2), HPO_4_^2−^ (7.2 < pH < 12.2), and PO_4_^3−^ (pH > 12.2). The phosphates could transform each other in the varied pH condition with the equilibrium constants through an equation of concentration partition as follows:

(1) $$H_{3}{\mathrm{PO}}_{4}\overset{K_{1}=7.50\times 10^{-3}}{\Leftrightarrow }\;H_{2}P{O_{4}}^{-}\overset{K_{2}=6.20\times 10^{-8}}{\Leftrightarrow }HP{O_{4}}^{2-}\overset{K_{3}=1.70\times 10^{-12}}{\Leftrightarrow }P{O_{4}}^{3-},$$

which indicates that the dissociated [PO_4_^3−^] is the lowest existing species in the pH condition of human body (7.3~7.8) [42]. The calcium exists as Ca^2+^ in acid condition with the pH up to 11.8, and then transformed as Ca(OH)_2_ at highly basic condition (Figure S2). In the calcium and phosphate combined Pourbaix diagram, the phase stable region of hydroxyapatite (Ca_5_(PO_4_)_3_(OH)) is pH > 8.9 at 37 °C, with the co-existence of dicalcium phosphate dehydrate (DCPD, CaHPO_4_·2H_2_O) between 4.7 < pH < 8.9 (Figure S3), which means the two species could transform each other with the stimuli of the environment in different mineralizer concentration conditions. Although the solubility of HA is the lowest in all kinds of calcium phosphate in the system of Ca^2+^-PO_4_^3−^-H_2_O at 37 °C [43], the surface of bone is covered by a thin layer of hydrated amorphous calcium phosphate [44], which is ready to transform into calcium-deficient and hydroxyl-deficient carbonated hydroxyapatite on the biomieralization epitaxy on bone surface and in the confined biological medium spaces [45]. Pourbaix diagram of Ca, P in aqueous condition indicates that the hydroxyapatite phase could only be stable when the pH is higher than 8.9. If the pH is lower than that value, the stable phase would be CaHPO_4_∙2H_2_O (3.5 < pH < 9.2) and Ca(H_2_PO_4_)_2_∙H_2_O (pH < 3.5). The stable region for all of the phosphate species shift to higher pH (e.g., PO_4_^3−^ from 12.2 to 12.5) when the temperature raise to 200 °C (for hydrothermal mineralization of HA and CHA), while that of calcium shift to lower pH (from 11.8 to 7.2) than that of at 37 °C (Figures S4 and S5). The combined diagram of Ca-P-H_2_O at 200 °C suggests that the stable region of HA shift to the pH as low as 6.8, with the co-existence of tricalcium phosphate (TCP, Ca_3_(PO_4_)_2_) and DCPD phases at the pH below 6.8 (Figure S6). Therefore, the reaction temperature and mineralizer amount should be carefully controlled to synthesize optimal hydroxyapatite materials.

**Figure 1:**
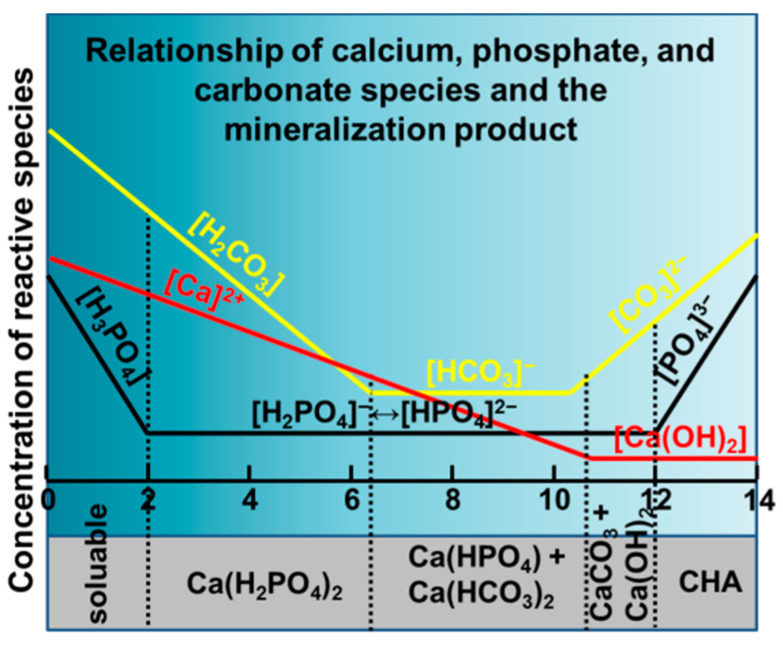
Reactive species of calcium, phosphate, and carbonate and the final solid phase products according to the thermodynamic data as calculated from Pourbaix diagram.

Carbonate exists as three species in aqueous condition at 37 °C, i.e., H_2_CO_3_ (hydrated CO_2_), HCO_3_^−^, and CO_3_^2−^, with the stable region of pH > 10.2 for CO_3_^2−^ anions (Figure S7), which also shift to higher pH (~10.5) at 200 °C (Figure S8). In the combined diagram, CaCO_3_ co-exists with HA at pH > 8.2 and pH > 6.8 for 37 °C and 200 °C, respectively (Figures S9 and S10). Because of the absence of thermodynamic data of carbonated-hydroxyapatites (CHAs), the co-existence region of CaCO_3_ and HA is predicted to be the most probable region for CHAs. Although calcite exists as the main impurity phase for the low crystallization temperature, it is ready to getting re-dissolved from the precipitation-dissolution balance to provide Ca-source for the further mineralization of apatite phase [46]. The reactive species at various pH regions in relation to the final mineralized phases were summarized in Figure 1 according to the systematic analysis of Pourbaix diagram. With increasing pH from 0 to 14, the most probable products were soluble solutions contained H_3_PO_4_, Ca^2+^, H_2_CO_3_ at pH < 2, amorphous Ca(H_2_PO_4_)_2_ or its hydrated crystal at 2 < pH < 6.2, Ca(HPO_4_) + Ca(HCO_3_)_2_ and their hydrated phases at 6.2 < pH < 10.3, CaCO_3_ + Ca(OH)_2_ and their hydrated phases at 10.4 < pH < 12, and carbonated hydroxyapatite (CHA) phases with varied compositions at pH > 12, respectively. Therefore, according to the Pourbaix diagram analysis, the carbonation of hydroxyapatite should be performed at an alkalinity higher than 12.

Based on the above Pourbaix diagram analysis, hydroxyapatite or even carbonated hydroxyapatite phases could be obtained at 37 °C spontaneously, as that of naturally grown bones and teeth, whereas amorphous calcium phosphate (ACP) is the most probable product as a precursor for poorly crystalized hydroxyapatite sandwiched between collagen fibers periodically [47]. For bone implant and repairmen materials, however, in most cases, the mineralization of apatite with high purity and crystallinity should be synthesized at higher temperature. Natural apatite minerals formed in volcano process (with high-temperature-high-pressure (HTHP) condition) in the deep sea that contained abundant soluble calcium and phosphate. The HTHP condition could provide high particle density of hydroxyapatite crystal nucleus, which results in small crystallite particles rather than large crystal [48], whereas HA crystals with micrometer size is unsuitable as bone tissue remedy materials in comparison with the nanometer sized samples [49]. Hydrothermal condition could provide adaptable temperature and pressure to simulate HTHP process with homogeneous control of the composition, morphology, and crystallinity of the products. The crystallization of hydroxyapatite depends on the supersaturation with the thermodynamic driving force defined by ln(1 + σ) = Δ*μ*/*k*T = ln(a/a_e_) [50], in which *μ* is the chemical potential in different states, k represents as Boltzmann constant, a and a_e_ are actual and equilibrium activities in crystallization, respectively. The chemical potential difference Δ*μ* in hydroxyapatite crystallization is defined as

(2) $$\Delta \mu =k\mathrm{Tln}\frac{{\left[a\left({\mathrm{Ca}}^{2+}\right)\right]}^{5}{\left[a({\mathrm{PO}}_{4}^{3-})\right]}^{3}\left[a\left({\mathrm{OH}}^{-}\right)\right]}{K_{sp}\left(hydroxyapatite\right)}$$

where *K_sp_* is the solubility product. The activities could be calculated according to Debye-Huckel theory as described in the literature [51]. In a definite reaction system with stoichiometric reactants of [Ca^2+^], [PO_4_^3−^], and [OH^−^], improvement of the reaction temperature could increase the Δ*μ* for the nucleation [21], and the reaction system with high density of nuclear results in the nanometer sized samples rather than to the growth of big crystals. Therefore, a moderate mineralization condition in hydrothermal route at the temperature of 200 °C was applied to synthesize the uniform nanometer sized carbonated hydroxyapatite samples with homogeneous composition.

### 2.2. Effect of Carbonate Source on the Structure of Carbonated Hydroxyapatite

**Figure 2:**
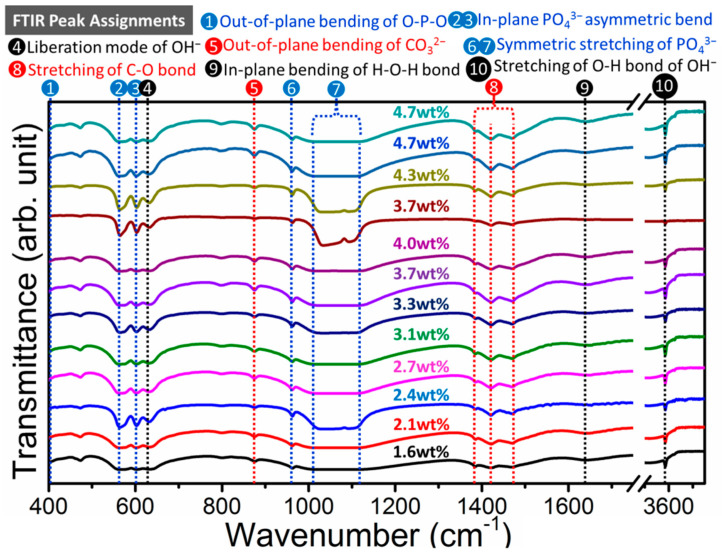
Fourier transform infrared spectroscopy (FT-IR) spectra of various carbonated hydroxyapatite samples with the CO_3_^2−^ amount varied from 1.6–4.7 wt% synthesized in hydrothermal condition by increasing the K_2_CO_3_ amount of 1.0–12.0 mmol in the starting reactants, respectively. Dotted lines are guided for the eye with notation of vibrations in various color round dots.

The carbonation of hydroxyapatite in hydrothermal condition is tunable with the starting concentration of K_2_CO_3_ in the reactant chemicals. We performed single factor variable experiment to address the influence of carbonate concentration on the carbonated-level in CHA samples that synthesized via mild hydrothermal route. The carbonation of hydroxyapatite in hydrothermal condition was mainly completed by adding K_2_CO_3_ as the sole carbonate source in the reactant. Samples with the same concentration of calcium, phosphate and the mineralizer amount were prepared by tuning the concentration of K_2_CO_3_ in the last procedure before hydrothermal reaction, with the amount of K_2_CO_3_ varied from 1 mmol up to 12 mmol. Fourier transform infrared spectroscopy (FT-IR) was applied to characterize the carbonization effect on the CHA products with varied carbonate concentration (Figure 2). All of the twelve spectra show the same sets of vibration mode for each absorption bands at nearly the same wavelength position (Table S1). The assignments of the FT-IR peaks are listed on the top of Figure 2. Characteristic vibrational modes of phosphate group (PO_4_^3−^) are clearly shown at 410 cm^−1^, 470 cm^−1^, 525–650 cm^−1^, 870 cm^−1^, and 960 cm^−1^, respectively [52]. Bands at 573 and 605 cm^−1^ are ascribed to the asymmetric bend mode of phosphate groups. Band at low wavenumber values could be assigned to the bending vibrations of O-P-O bond. Absorption peak at 960 cm^−1^ is ascribed to symmetric stretch of PO_4_^3−^ tetrahedral units. Wide absorption band at 1000–1200 cm^−1^ may be ascribed to asymmetric stretching vibrations of P-O band. Shoulder band at 631 cm^−1^ is ascribed to the liberation band of OH^−^. The transmission peak at 3571.7 cm^−1^ is ascribed to the stretching vibration mode of OH^−^ in hydroxyapatite. A small absorption band at 1630 cm^−1^ for the carbonated hydroxyapatite samples could be ascribed to the bending mode of absorbed H_2_O molecule at the surface of nanorod particles. Bands at about 2342 cm^−1^ is assigned to the characteristic absorption of CO_2_ in atmosphere [53]. Characteristic bands of carbonate in CHA samples occur in the regions of 1400–1600 cm^−1^ of asymmetric stretch vibration, 870–880 cm^−1^ for bend vibration of our-of-plane mode. Peaks at 1456 cm^−1^ and 874 cm^−1^ are characteristic peak of carbonate CO_3_^2−^ ions of B-type CHA [54]. Symmetrical doublet bands at 1421 cm^−1^ and 1473 cm^−1^ are associated with the carbonate CO_3_^2−^ ion in the single structural environment for B-type CHA, which is the characteristic signal of the planar carbonate anions substituted the crystallographic site of tetragonal phosphates. Inevitable existence of AB-type remains in the materials synthesized via high energy milling method up to 100 h [55]. The transmittance intensity of carbonate vibrations at 1421 and 1471 cm^−1^ decreased with the incretion of K_2_CO_3_ amount, indicating the carbonate content is increased gradually from 1.6 wt% to 4.7 wt%. The carbonate content has been determined by Clasen’s method by integrating the area of carbonate peak at 1421 cm^−1^ and phosphate peak at 605 cm^−1^, respectively [56]. As demonstrated in Figure 2, increasing the started ratio of carbonate in the reactant could improve the final ratio of carbonate in the as-synthesized materials. Therefore, increasing the carbonate concentration in the reactant in hydrothermal condition could promote the carbonation level in the final CHA products. Besides, the carbonate ratio for the as-synthesized samples could meet the range of carbonate ratio in human enamel.

**Figure 3:**
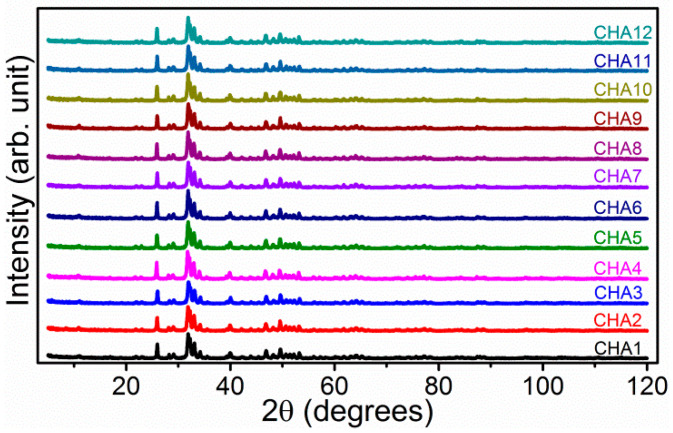
Powder X-ray diffraction (PXRD) data of hydrothermally synthesized carbonated hydroxyapatite (CHA) samples with varied amounts of K_2_CO_3_ for the starting reactant in the same synthetic procedure: CHA1 1.0 mmol (0.1382 g), CHA2 2.0 mmol (0.2764 g), CHA3 3.0 mmol (0.4146 g), CHA4 4.0 mmol (0.5528 g), CHA5 5.0 mmol (0.6910 g), CHA6 6.0 mmol (0.8292 g), CHA7 7.0 mmol (0.9674 g), CHA8 8.0 mmol (1.1056 g), CHA9 9.0 mmol (1.2438 g), CHA10 10.0 mmol (1.3820 g), CHA11 11.0 mmol (1.5202 g), and CHA12 12.0 mmol (1.6584 g), respectively.

Phase purity and crystallographic information of the as-synthesized series of CHA samples were measured with powder X-ray diffraction (PXRD) method (Figure 3 and Figure S11). The diffraction peaks for the CHA samples with different K_2_CO_3_ amounts in the reactants are nearly at the same positions, indicating the samples were crystalized in the same structure without significant effect on the lattice for the carbonate-substitution. All of the PXRD data were simulated via Rietveld refinement with a primary Na-doped B-type carbonated hydroxyapatite crystallographic model of ICSD card No. 92,322 as the nominal chemical formula of (Ca_3.36_Na_0.08_)(Ca_5.04_Na_0.72_)(PO_4_)_3.6_(CO_3_)_2.4_(OH)_2_ [57]. All of the diffraction peaks could be indexed into a hexagonal space group of P63/m (No.176). No additional peaks could be detected in the PXRD patterns for each CHA sample, indicating that all of the products are phase pure, without any secondary impurity in the hydrothermal products. The residual parameters wR_p_, R_p_, and reduced χ^2^ are slightly higher than the expected refinement results (Table S2), which could be attributed to the relative poorer crystallinity of carbonated hydroxyapatite nanocrystals than that of un-substituted apatite phases. The calculated unit cell parameters of a decreased and c increased with the increasing carbonate substitution level of phosphate in the hydroxyapatite crystal lattice, with a nearly unchanged cell volume for all of the as-synthesized CHA samples due to the synergetic effect of a- and c-axis. The lattice parameters change from a = 9.4207(7) Å and c = 6.8859(5) Å for CHA1 (Figure S11a) to a = 9.4089(8) Å and c = 6.9027 (9) Å for CHA12 (Figure S11l). Although the lattice parameters are not linearly evolved with the K_2_CO_3_ amount in the starting reactants, continuous decrease for a-parameter and increase for c-parameter could be found in the refinement results (Table S2), which indicates that improvement of starting carbonate concentration is effective to improve the CO_3_^2−^ ratio in the hydrothermally synthesized CHA samples. Site occupancy of P/C ratio was refinement after all of the other parameters have been optimized, and the site of P + C was set to be 1 in confinement with the same crystallographic site of x, y, and z and isotropic thermal vibration parameter of U_iso_ (Table S3). The ratio of P/C decrease from 0.955(5)/0.045(5) for CHA1 to 0.780(11)/0.220(11) for CHA12, indicating the carbonate ratio has been improved in high K_2_CO_3_ concentration hydrothermal conditions. This result is in agreement with that of FT-IR spectra in Figure 2. Crystallite size of as-synthesized RE-doped HA was estimated by Scherrer’s equation: R_hkl_ = Kλ/β_0_cosθ, where R_hkl_ indicates the crystallite radii that perpendicular to the reflection crystallographic plane (with its Miller index of hkl) with a unit of nm; θ is the diffraction Bragg angle (unit: degree); λ is the wavelength of the copper source X-ray of Kα line (unit: nm), which equals to 1.5406 Å for Kα1 of copper target; β_0_ is the peak width at the half maximum (unit: rad); K defined as shape factor according to the crystallite, which also determined by the definition of β_0_ and R_hkl_, and in this case K = 0.9. The average crystallite size of CHA is calculated as 54–74 nm, which is in agreement with the result of SEM graphs (Table S4).

### 2.3. Structure Characteristics of B-Type Carbonated Hydroxyapatite

**Figure 4:**
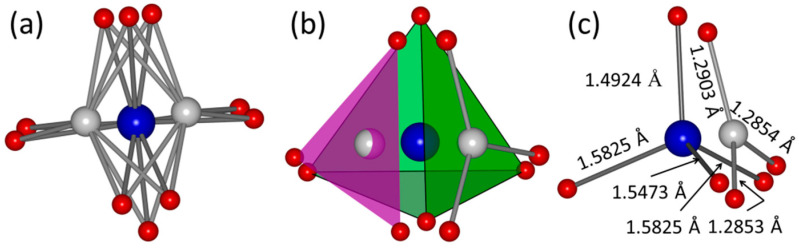
Local structure of carbonate-substituted hydroxyapatite. (**a**) The multiplicity of P/C (6 h-site) and oxygen (6 h- and 12 i-sites), (**b**) Assumed stack configuration of CO_3_^2−^ planar and PO_4_^3−^ tetrahedral units at the same crystallographic site, (**c**) relative position and bond length of CO_3_^2−^ and PO_4_^3−^ units.

The carbonate in hydroxyapatite has two site selective possibilities either at that of OH^−^ site or at that of PO_4_^3−^ site, which results in A-type and B-type substitution as discussed in the introduction. Both types of carbonation are charge unbalanced substitution, which should be compensated by the formation of cationic defects by either doping other cations with higher (for OH^−^) or lower (for PO_4_^3−^) charges, or formation of Ca^2+^ deficiency. All of the above substitution routes lowered the symmetry at definite crystallographic sites and the crystallinity than that of un-substituted hydroxyapatite crystals. By summarize the previous literature on the crystal structure of carbonated hydroxyapatites, we found that A-type CHA is generally crystalized at high temperature condition with adequate carbonate sources during their mineralization processes. B-type CHA is stable in aqueous solutions via the formation of proton-related defects to stabilize the charge difference between CO_3_^2−^ and PO_4_^3−^. The substitution results in the charge and coordination difference, which reflected in the crystal structure as non-stoichiometric occupancy of oxygen atoms (Figure 4a). Although all of the possible atomic positions have been plotted, oxygen atoms at the very near site occupy the site by spontaneous probability depending on the central atom of either P or C. The substitution of triangle planar CO_3_^2−^ anions for tetrahedral PO_4_^3−^ by replacing one of the four planes of the tetrahedral unit forming a sloping face with an angle of CO_3_^2−^ plane to c-axis of 30° [58], with a slight rotation of O-O-O plane between the face of PO_4_^3−^ and CO_3_^2−^ plane (Figure 4b). This rotation of the CO_3_^2−^ group is attributed by the response of charge imbalance to the rigid structure of Ca-O-P framework in the overall hydroxyapatite structure. Although the theoretical chemical bond length of C-O and C=O is 1.43 Å and 1.23 Å, the average bond length between C and O atoms in H_2_CO_3_ molecule is 1.36 Å due to the delocalization of electrons by forming Π_4_^6^ hybrid orbitals [59], which will be further getting shrinkage in the crystallographic structure due to the loss of attraction of H^+^. In the CHA structure, the bond length of C-O is 1.2854 Å, 1.2853 Å, and 1.2903 Å. The relative longer for the erected C-O bond is attributed to the attraction of Ca^2+^(1) at the neighbor site (Figure 4c). Molecular structure of PO_4_^3−^ is formed by nonequivalent hybridization of P ([Ne]3s^2^3p^3^) atom with five electrons in four sp^3^ orbitals, which results in three long P-O bond length of ~1.55 Å and one short P-O bond length of ~1.49 Å. The substitution of B-type carbonated hydroxyapatite could reach to a high degree via this structure construction configuration.

### 2.4. Morphology Evolution from Hydroxyapatite (HA) to Carbonated Hydroxyapatite (CHA)

**Figure 5:**
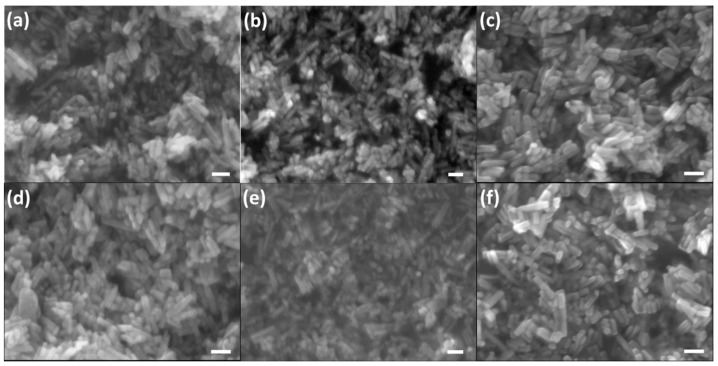

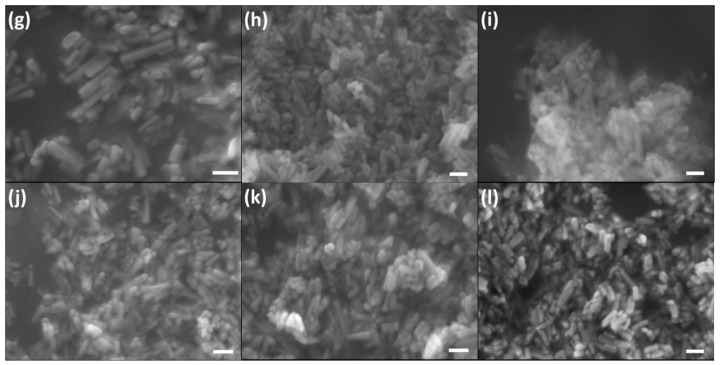
SEM graphs of carbonated hydroxyapatite nanocrystals with the K_2_CO_3_ amount in the reactants of (**a**) 1.0 mmol, (**b**) 2.0 mmol, (**c**) 3.0 mmol, (**d**) 4.0 mmol, (**e**) x = 5.0 mmol, (**f**) 6.0 mmol, (**g**) 7.0 mmol, (**h**) 8.0 mmol, (**i**) 9.0 mmol, (**j**) 10.0 mmol, (**k**) 11.0 mmol, and (**l**) 12.0 mmol, respectively. Scale bars in all of the SEM graphs are 100 nm.

Morphology evolution of CHA nanocrystals with different carbonate level were characterized in scanning electron micrographs (Figure 5). All of the as-synthesized CHA samples crystalized as nanorod shape with uniform distribution. Average sizes of the as-synthesized hydroxyapatite nanorods are in the range of 20–40 nm in the diameter, and 70–200 nm in length, which approaches the native biologically derived morphology of the inorganic components in enamel of human teeth [12]. The variation of the particle sizes is getting increased in higher carbonate concentration in the reactants, which could be attributed the inhomogeneity in the localized nucleation and fast growth of the nanocrystals in hydrothermal condition. The average length-to-width ratio of as-synthesized CHA nanocrystals decreases gradually from 3.6 to 3.0 with the increasing substitution content from the starting K_2_CO_3_ amount of 0.1382 g (0.1 mmol) to 1.6584 g (1.2 mmol). The morphology evolution of CO_3_^2−^ substituted crystals with increasing K_2_CO_3_ in starting reactants is in agreement with the crystallographic results according to Rietveld refinement of XRD data (Table S4). The more carbonate in the crystals, the higher degree of a-axis elongation and c-axis contraction, i.e., low length/diameter (L/D) ratio. This effect has also been found in Na-participated mineralization process with varied carbonate contents [60]. The formation of the hexagonal rod shape of hydroxyapatite has been analysed according to Bravais–Friedel–Donnay–Harker (BFDH) theory in our previous work [38]. The ideal BFDH morphology of Ca_10_(PO_4_)_6_(OH)_2_ crystal is hexagonal prism enclosed by {110}, {−210}, {1–20}, and {001} facets. The preferential growth of hydroxyapatite is usually along c-axis, which is similar as that of in bone-tissue [61]. Carbonated-hydroxyapatite samples prepared by precipitation method with urea as the carbonate source were crystalized as large crystals with the sizes up to 300 nm [23], which could be further increased (to 60–116 μm) by lowering the hydrolysis rate via acetamide substitution to provide low supersaturation of mineralizer concentration [62]. In the urea-assisted syntheis of carbonated hydroxyapatite, the shape of apatite crystals changes from long nanorod with high length-to-radius ratio to the short rod with increasing the amount of urea, which indicates a retarding effect on the crystal growth along c-axis while propagation enhanced along a-axis [63], which is similar as that of various hydroxyl-tuned the crystal shape of CHA [64]. The reason for this phenomenon could be ascribed the incorpration of CO_3_^2−^ into the apatite crystal lattice and possible NH_4_^+^ guided growth of the PO_4_^3−^ through hydrogen bond electrostatic interactions at the newly formed surface after nucleation. Hydroxyapatite synthesized at low temperature via aqueous route is prone to form large crystals, which may be resulted from the low dehydration rate produced less nucleate in the reaction system that stimulate the increasing growth of the crystals gradually. Samples obtained at 120 °C hydrothermal condition result in long wiskers [65]. In lower temperature of 100 °C, the hydroxyapatite could grow as 50–200 nm wide, and hundreds of micometers long patterned crystals with the assistance of gelatin [66]. The introduction of ethylene diamine tetraacetic acid (EDTA) group in hydrothermal crystallizaiton of hydroxyapatite results in dendrite-like clustered nanorods of AB-type CHA [53]. With the assistance of urease, <001> oriented plate-shape HA has also been synthesized by providing basic mineralizer condition of urea hydrolysis [67]. Sharp faceted, micrometer-sized hydroxyapatite samples with hexagonal rod morphology could be obtained via hydrothermal method at low temperature of 90 °C [68]. Large hydroxyapatite crystals with high purity were proved that difficult to decomposed by osteoclast cells that retards the promotion in osteoblast as continuous supplier of new resources for bone formation. For the carbonated hydroxyapatites, factors of low reactant concentration, low pH, and low temperature could also produce larger crystals with well-defined morphologies [69]. Supersaturation of the reactant system also affects the morphology of HA in hydrothermal condition. The addition of urea and glutamic acid in the reaction system results in microspheres composed of small flakes of HA at stoichiometric of the reactants, which changes into micrometer size hexagonal rod at high phosphate ratio [70]. Polymers with amino (-NH_2_) groups, such as polyaspartic acid, works as template for crystallization of hydroxyapatite with hierarchical morphologies [71]. Small molecule with multiple hydroxyl groups inhibit the crystallization in a classical pathway and results in small pieces of organized aggregation [72,73]. Therefore, the carbonated ratio in CHA not only affects the structure of the samples, but also changes the morphology slightly according to SEM analysis.

### 2.5. Rare-Earth Ions Substitution in HA and CHA Materials

Doping of rare-earth in hydroxyapatite crystal lattice should also take the thermodynamic factors into consideration. According to the Pourbaix diagram, the rare-earth could be existed in two stable regions, i.e., RE^3+^ and (oxy)hydroxide (REO(OH) or RE(OH)_3_). Synergic effect is the most crucial to achieve a successful doping. As in the case of rare-earth doping in hydroxyapatite and carbonated hydroxyapatite, the choice of phosphate, pH of the system, and the hydrothermal reaction temperature are the main factors. Let’s take the doping Tb in CHA as an example to analyze the suitable synthetic condition. In the pH < 6.9, terbium exists as aqueous Tb^3+^ ions at 37 °C, which is prone to combine OH^−^ and forms Tb(OH)_2_^+^ aqueous cations at 6.9 < pH < 8.25, and changes into partially dehydrated product of TbO(OH) at the pH higher than 8.25 (Figure S12). When the same concentration of terbium solution is kept at 200 °C, the dehydration occurs to low pH region (Figure S13), indicating that it does not need that much of mineralizer to be incorporated into the crystal lattice of hydroxyapatites. Other rare-earth cations show a similar thermodynamic behavior as that of Tb^3+^, i.e., the main existence state is RE^3+^ below pH = 7, and gradual formation of rare-earth (oxy)hydroxides with increasing pH. However, it is difficult to dehydrate for rare-earth hydroxides by merely increasing the pH, which should be assisted by temperature or pressure of the reaction system. Therefore, the doping of RE^3+^ into hydroxyapatite crystal lattice at room temperature is impossible. The improving reaction temperature although reduced the dehydration probability, however, the sole temperature effect induces the dehydration of RE^3+^ and Ca^2+^ and phosphate anions respectively, rather than formation of a single phase crystalline product. Especially, hydroxyapatite has two crystallographic sites for Ca, and therefore two choices for the doped RE^3+^ ions. Generally, RE^3+^ ions with the larger ionic radii are prone to dehydrate at lower pH, especially in hydrothermal condition, i.e., dehydration is easier that assisted by pressure [74,75]. Therefore, it should be delicately control the pH and reaction temperature for RE^3+^ involved hydrothermal synthesis, especially for the competition phase could be prone to form, such as hydroxides or (oxy)hydroxides.

**Figure 6:**
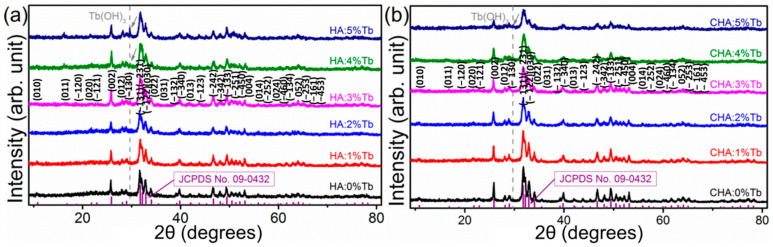
Powder X-ray diffraction (PXRD) results of hydrothermally synthesized hydroxyapatite samples: (**a**) PXRD of various concentration of Tb-doped hydroxyapatite (HA:x% Tb) samples; (**b**) PXRD of various concentration of Tb-doped carbonated hydroxyapatite (CHA:x% Tb) samples. Purple bars indicate the theoretical peak positions and intensities of a hydroxyapatite from JCPDS Card No. 09-0432. Grey dash lines indicate the impurity phase peak position of Tb(OH)_3_ for the high doping level samples.

To avoid the crystallite and strain effect, K_2_CO_3_ was used as the sole carbonate source in the syntheses of Tb^3+^-substituted HA and CHA samples. Structure of all as-prepared RE-doped hydroxyapatite materials was determined by a laboratory powder X-ray diffraction technique, which was simulated by Rietveld fitting method. All of the diffraction peaks could be well-fitted either in peak positions or intensities, which indicates pure phase of the samples obtained via hydrothermal method. As indicated by the theoretical diffraction peak positions that marked with green bars, all of the five Tb^3+^ doped samples could be ascribed into the same set of diffraction peaks, indicating the successful doping of Tb^3+^ in the crystal lattice of hydroxyapatite (Figure 6). With increasing the doping level of Tb^3+^, an additional diffraction peak was found in the PXRD patterns, which implies that a secondary impurity phase of Tb(OH)_3_ has been produced with hydrothermal method in this condition. Slightly mismatch of the theoretical fitting and observed diffraction peaks at 2-theta of 26.0° and 31.9° indicates the existence of preferential orientation at the {002} and {−131} crystallographic planes. The existence of preferential orientation for the samples may be resulted from the geometry shape of as-obtained nanocrystals as well as the native self-organization of them. The refinement results indicate that the cell parameter a decreased, while c increased in respect to the pure HA without any substitution, which is in agreement of the difference between biological and mineral apatite [76]. For the high doping level in the case of Tb-doped HA and CHA, impurity peak of Tb(OH)_3_ phase could be detected in the XRD pattern at the doping ratio > 4% and 5% for HA and CHA, respectively. The highest doping level of Tb^3+^ is lower than that of Eu^3+^, which may be resulted from the smaller ionic size of Tb^3+^ in the six-coordinated state that much lower than Ca^2+^ in comparison with that of Eu^3+^. The relative higher doping level of Tb^3+^ in CHA than that of HA may be resulted from the higher defect formation energy in carbonated HA with the modifying effect on bond length and coordination of Ca [77]. For Tb-substituted HA and CHA, the doping of Tb does not change the morphology of the apatite phase nanocrystals, which may due to the doping level is too low to make a contribution to the crystallization process. When the doping ratio is over 5%, long rod shape Tb(OH)_3_ could be observed with higher contrast than that of HA and CHA nanorods, indicating the doping is unsuccessful for the high doping levels (Figure S14). Powder X-ray diffraction results indicate other RE^3+^ of Pr^3+^, Sm^3+^, and Ho^3+^ could also dope into the crystal lattice of hydroxyapatite (Figures S15–S17). All of the diffraction peaks could be indexed in accordance with JCPDS Card No. 09-0432, and no secondary phases could be found in the XRD patterns, indicating the doping is also successful for these rare-earth elements.

Because of the contraction of lanthanides, effective ionic radii for RE^3+^ reduced gradually with increasing atomic number in element periodical table (Table 1), which leads to higher charge density than that of Ca^2+^ in the six-coordinated configuration of hydroxyapatite crystal lattice. In our case, the radii of Pr^3+^(VI) is 99 pm, near to that of Ca^2+^ in six-coordinated configuration. However, the higher charge density of Pr^3+^ should be taken into consideration in either crystallization process or analysis the photoluminescence mechanism. All the radii of RE^3+^ with RE = Pr, Sm, Eu, Tb, and Ho are slightly smaller than that of Ca^2+^. Ca1 is six-coordinated, with an ionic radius of 100 pm, while Ca2 is a seven-coordinated site, and the ionic radius is 106 pm. The radius of Eu^3+^ in 6-coordinated state is 94.7 pm, and that in 7-coordinated state is 101 pm. Although the existence of ionic radii and charge density difference between RE^3+^ and Ca^2+^, RE^3+^ substitution of Ca^2+^ shows high tolerance, especially for the compound that contained valence change transition metals [78]. Bond length of Ca1-O is longer than that of Ca2-O in average in hydroxyapatite, and the theoretical bond length difference of RE-O shows longer for the RE of La-Nd, while shorter for that of Sm-Lu [79]. Therefore, the doping of RE in hydroxyapatite could be affected by the crystal field of Ca^2+^ in its crystallographic site.

FT-IR spectra of hydrothermally synthesized RE^3+^ with different RE^3+^ and doping amounts were shown similar absorbance bands as that in CHA (Figure S18) and no bands related to RE^3+^ could be assigned, indicating the doping of RE^3+^ into the crystal lattice. Morphology of Sm^3+^ and Ho^3+^ doped hydroxyapatite nanocrystals with various doping level was shown in Figure S19. The morphology of as-synthesized hydroxyapatite nanocrystals was radius-uniformly distributed hexagonal rod with a length-to-diameter ratio of ~2.7. As discussed in the structure part of HA, all of the RE^3+^-doped HA samples are crystalized as the same space group of HA, i.e., P6/m. The crystal sizes are reduced with increasing doping concentration of Ho^3+^, which indicates the introducing of Ho^3+^ in hydrothermal reaction system changes the crystallization mechanism after nucleation of hydroxyapatite. Increasing doping level of Ho^3+^ results into smaller sized crystals, which is difficult to discriminate the definite shape in SEM graphs.

Composition of the HA, CHA, and rare-earth doped samples were assessed by ICP-AES and EDS methods accordingly (Table 2). With increasing the doping content of Tb^3+^ in the reactant, the Tb^3+^ ratio in the hydroxyapatite and carbonated hydroxyapatite increased simultaneously. These results could be clearly discriminated from the EDS spectra graphs in Figure S20. All of the measured Tb-content in the samples is lower than that of theoretical values, which could be attributed the dissolution–precipitation balance of Tb-species in the reactive solutions. The relative higher of the doped Tb^3+^ in the products of CHA series could be attributed to the electrostatic effect and lattice distortion due to the incorporation of CO_3_^2−^ in the structure. The measured value of EDS results is lower than that of ICP-AES results for all of the HA and CHA series, which may be resulted from either the difference of the measurement method or the adsorption of trace amount of Tb^3+^ ions in the solvent media for the dissolution process or the slightly vaporized of solvent.

### 2.6. Photoluminescence of Tb^3+^ in the Site-Selective Occupancy of Hydrothermally Crystalized HA Nanomaterials

**Figure 7:**
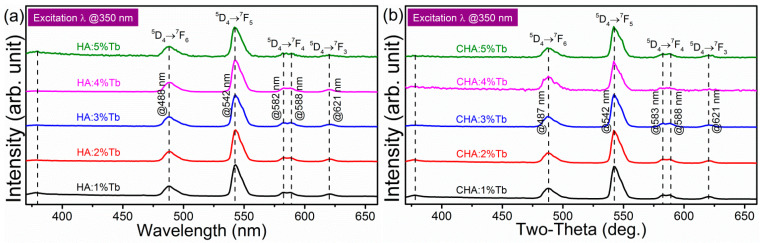
Emission spectra of Tb^3+^ in (**a**) hydroxyapatite (HA), and (**b**) carbonated-hydroxyapatite (CHA) nanocrystals with various doping levels from 1% to 5%.

Excitation spectra of Tb^3+^ in CHA lattice was collected to monitor the emission at 452 nm as shown in Figure S21. Peaks at 302, 318, 342, 350, 360, 368, and 376 nm could be discriminated from the excitation spectrum, indicating the energy transitions of the multiplets ^5^H_6_, ^5^H_7_, (^5^G_2_,^5^L_6_), (^5^G_4_,^5^L_9_), ^5^G_5_, ^5^L_10_, and (^5^D_3_,^5^G_6_), respectively [81]. The peak at 284 nm is attributed by forbidden transition 9D levels of 4f^8^→4f^7^5d^1^ transition [82]. Room temperature emission spectra of Ca_10_(PO_4_)_6_(OH)_2_:Tb nanophosphors were shown in Figure 7. Broad bands at 355 nm could be ascribed to the O^2^–Tb^3+^ charge transfer band (CTB), as noted as dotted lines in the figures. Theoretically, the higher energy ^5^D_3_ spectroscopic level could also be luminescent, but it fast relaxed to ^5^D_4_ via the vibration of ligands and therefore usually be neglected the contribution of the transition from this band. Besides, the existence large amounts of hydroxide (OH^−^) anions in the crystal lattice could act as high-energy phonons that quenches ^5^D_3_ energy level rapidly via non-radiated scattering [82]. Therefore, the emission peak of ^5^D_3_–^7^F*_j_* (*j* = 0–6) had not been detected, different from fluoride ligand host of K_2_Y_2_F_5_ [83] and oxide host of Lu_2_SiO_5_-Gd_2_SiO_5_ solid solution single crystal [84]. The emission of Tb^3+^ in the crystal field is dominated by all of the possible transitions of ^5^D_4_–^7^F*_j_* (*j* = 0–6), however, the transitions of ^5^D_4_–^7^F*_2_*, ^5^D_4_–^7^F*_1_*, and ^5^D_4_–^7^F*_0_* are very weak and could not be detected by the spectrometer. Photoluminescent emission peaks at 370–660 nm can be ascribed to the internal electron transfer with characteristic configurational 4f^8^–4f^8^ transitions of Tb^3+^ in the hydroxyapatite host lattice: ^5^D_4_–^7^F*_j_* (*j* = 3–6). Peaks at 488, 542, 582–588, and 621 nm could be assigned to the energy transition of ^5^D_4_–^7^F_6_, ^5^D_4_–^7^F_5_, ^5^D_4_–^7^F_4_, and ^5^D_4_–^7^F_3_, respectively [85]. These results indicate that all of the Tb^3+^ in HA lattices were coordinated by six oxygen atoms in the Ca1-site. With increasing the doping level of Tb in HA, the peak positions of emission spectra keeps the same, but getting lower in the intensity, indicating the high doping level induced fluorescence quenching due to the cross-relaxation and energy transfer in the high concentration of activator center in hydroxyapatite lattice. Tb^3+^ in carbonated-hydroxyapatite (CHA) lattices shows similar emission spectra as that in HA (Figure 7b). The peak positions of ^5^D_4_–^7^F*_j_* (*j* = 3–6) transitions are nearly the same position as that of HA. Quenching effect is also found at the Tb^3+^ concentration over 4%. Concentration quenching could be explained by the two main models: Exchange interaction and multipole-multipole interaction. Critical energy transfer distance (R_c_) could be estimated by the Equation:

(3) $$R_{c}=2{\left(\frac{3V}{4{\pi X}_{c}N}\right)}^{\frac{1}{3}},$$

where V is the cell volume of HA/CHA, X_c_ is the critical activator concentration, N is the total number of activated site within the unit cell [86]. For the case of CHA:1 Tb%, V = 530.3 Å^3^, X_c_ = 0.01, N = 2, therefore, R_c_ is calculated as 37.00 Å. Since R_c_ > 9.4 Å of the unit cell length, the energy transfer for the Tb^3+^ ions can be ascribed to multipole-multipole interactions. For the case of Pr^3+^ doped samples, the theoretical emission bands should be found at 487, 530, 604, 620, 648, 706, and 728 nm for the emission of ^3^P_0_–^3^H_4_, ^3^P_0,1_–^3^H_5_, ^1^D_2_–^3^H_4_, ^3^P_0_–^3^H_6_, ^3^P_0_–^3^F_2_, ^1^D_2_–^3^H_5_, and ^3^P_0_–^3^F_3_ + ^3^F_4_, respectively [87]. In our Pr^3+^ doped samples, although most of the theoretical emission peaks could also be observed (Figure S22), no separated peaks could be found, which could be ascribed to the internal energy transitions resulted from of thermally induced expansion of emission cross section of Pr^3+^ according to Judd-Ofelt theory [88]. Similarly, only two weak emission peak of 601 and 640 nm of Sm^3+^-doped CHA could be observed, which are ascribed to the emission of ^4^G_5/2_–^6^H_7/2_ and ^4^G_5/2_–^6^H_9/2_, respectively [89]. Ho^3+^ is an up-conversion emission center [27], which could not emit visible light wavelength signals under the excitation of ultraviolet excitation sources and therefore no emission peaks have been observed. As in our case, the luminescent properties and its potential effect in carbonated hydroxyapatite will be studied in the next step. Decay curves for these RE-doped carbonated hydroxyapatite samples have also been collected, however, all of them could not be fitted with a double-exponential function as that of in Eu^3+^- and Tb^3+^-doped samples, which could be ascribed the weak emission peaks in their emission spectra.

Decay curves of Tb-doped HA and CHA with various doping levels were shown in Figure S23. We monitored all of the possible decay at peaks for the energy transition of ^5^D_4_–^7^F_6_ (488), ^5^D_4_–^7^F_5_ (542), ^5^D_4_–^7^F_4_ (582–588), and ^5^D_4_–^7^F_3_ (621) nm, respectively. All of the decays could be fitted by a double-exponential function of I = I_0_ + A_1_exp(−t/τ_1_) + A_2_exp(−t/τ_2_), where I is the intensity of the luminescence at each transition peak, I_0_ represents the background luminescence intensity, A_1_ and A_2_ are fitted parameters with constant values, τ_1_ and τ_2_ are the decay times for two respective processes. The average lifetimes for each transition was calculated by the equation of τ = (A_1_τ_1_^2^ + A_2_τ_2_^2^)/(A_1_τ_1_ + A_2_τ_2_). The lifetimes of the Tb-doped samples are in the range of 3.5–5.4 ns for all of the allowable transitions (Table S4), indicating the fast electron transition in these orbitals, which could not been affected by the substitution of carbonate at the phosphate sites. The average τ increased from 1% to 3% of Tb doping in HA and CHA lattice, and then decreased, which could be ascribed concentration induced quenching and the contribution of impurity phase of Tb(OH)_3_, indicating the energy transfer between Tb^3+^-Tb^3+^ ions at high doping amounts [90].

### 2.7. Cytotoxicity Assessment of As-Synthesized HA, CHA, and RE-Doped HA, CHA Nanomaterials

**Figure 8:**
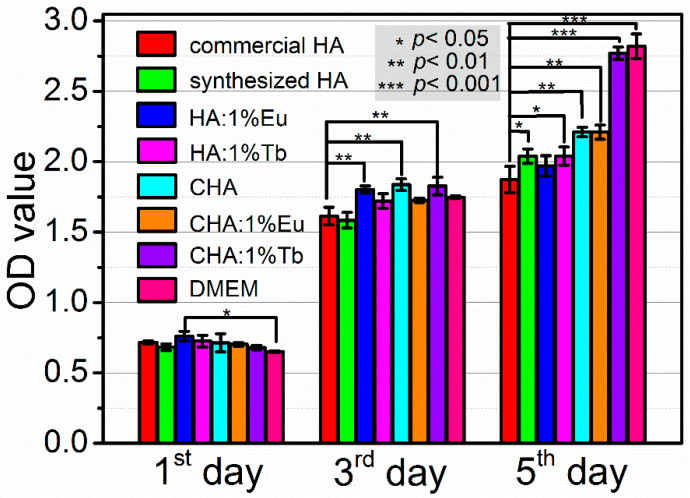
Cell attachment and proliferation in the extracts of various apatite materials: Commercial HA, as-synthesized HA, CHA, and Eu^3+^- and Tb^3+^-doped HA and CHA samples, respectively.

Cell viability and proliferation of MC3T3-E1 cells in the extracts were examined by MTT assay for the synthesized HA, CHA, and RE^3+^-doped HA, CHA materials, in comparison with that of commercial HA and DMEM (Figure 8). All of the materials show no cytotoxicity, and the synthesized HA and CHA materials show superior proliferation performance than that of commercial HA. There were no statistical differences between the synthetic materials with commercial HA within 24 h. Higher OD values were found in Eu^3+^ and Tb^3+^ doped HA samples, and all CHA samples since the 3rd day. Statistical significance of *p* < 0.01 between commercial HA and HA:1%Eu, CHA, and CHA:1%Tb was found in the 3rd day proliferation results, which increased the significance to *p* < 0.001 for CHA:1%Tb at the 5th day. The experiments in CHA and CHA:1%Eu also show significant value of *p* < 0.01. These results indicated that the carbonation of hydroxyapatite could promote cell proliferation than that of commercial HA and synthesized HA materials, especially for the 1%Tb doped CHA sample.

## 3. Materials and Methods

### 3.1. Chemicals

Chemicals of KH_2_PO_4_·2H_2_O (99.0%), Ca(NO_3_)_2_·4H_2_O (99.0%), Pr(NO_3_)_3_ (99%), Sm(NO_3_)_3_ (99%), Eu(NO_3_)_3_ (99%), Tb(NO_3_)_3_ (99%), Ho(NO_3_)_3_ (99%), and KOH (85%) were purchased from Sinopharm Chemical Reagent Co. Ltd. (Shanghai, China) Analytical grade of K_2_CO_3_, urea (CO(NH_2_)_2_) were purchased from Beijing Chemical Industry (Beijing, China). All of the chemicals were used without further purification. Solutions of 0.4 M KH_2_PO_4_, Ca(NO_3_)_2_ and RE(NO_3_)_3_ were prepared by dissolving the as-obtained chemicals in deionized water (>18.2 MΩ) in advance. All of the reactants and mineralizer chemicals were used potassium salt or hydroxide to keep the reactive condition of K^+^ environment to avoid the interruption of Na^+^ for the formation of carbonated products and avoided the effect of RE^3+^ doping in (carbonated-)hydroxyapatite crystal lattice via charge balance. To avoid the influence of dissolved CO_2_ on the formation of carbonated hydroxyapatite, the deionized water was boiled for 10 min to reduce the carbonate fraction in the reactant solvent, because of the lower solubility of carbon dioxide in aqueous medium at high temperature [91].

### 3.2. Hydrothermal Syntheses of the Samples

Because Na^+^ is prone to incorporate into the crystal lattice of hydroxyapatite, to avoid the effect of Na^+^ on the carbonation, RE-substitution and site-selective occupancy, all of the salt that could contain alkaline cations of the phosphate source, carbonate source, and the mineralizer are K^+^-type, i.e., KH_2_PO_4_, K_2_CO_3_, and KOH, respectively. To avoid the evolvement of halide, only the nitric salt of calcium and rare-earth sources were used, i.e., Ca(NO_3_)_2_ and RE(NO_3_)_3_.

#### 3.2.1. Synthesis of Hydroxyapatite Nanocrystals.

The synthesis of Ca_10_(PO_4_)_6_(OH)_2_ nanocrystals was performed in a Teflon-lined autoclave with capacity of 50 mL, followed by our previous work with minor modifications [38]. Firstly, 12.6 mL KH_2_PO_4_ and 21.0 mL Ca(NO_3_)_2_ solution were mixed in the beaker with continuous magnetic stirring for 10 min. A translucent mixture for the starting chemicals was formed stoichiometrically. The KOH mineralizer was added into the mixture with a fixed amount of 4.5 g. The whole mixture was poured into the Teflon-lined stainless steel autoclave after the beaker cooled down to room temperature. Then, the autoclave was transferred into an oven of 200 °C for continuous heating for 24 h. White precipitates were obtained in the bottom of the autoclave, which have been washed by deionized water for several times until the pH of the products reaches to 7–8. The pasty products (due to the absorption of solvent water between the nanoparticles) were dried at 80 °C overnight and stored in a self-sealed plastic tube for further measurement. The crystallization of HA followed the reaction equation as-shown in the following reaction:

10Ca(NO_3_)_2_ + 6KH_2_PO_4_ + 14KOH → Ca_10_(PO_4_)_6_(OH)_2_ + 20K^+^ + 20NO_3_^−^ + 12H_2_O (4)

#### 3.2.2. Synthesis of Biomimetic Carbonated-Hydroxyapatite

The synthesis of carbonated-hydroxyapatite materials follows a similar protocol as that of HA with the addition of carbonate source in the reactants. The carbonation of hydroxyapatite was performed in the reactant mixing process, in which K_2_CO_3_ and urea with different concentration were used as the carbonate source, respectively. To explore the concentration of carbonate source effect on the carbonation level in the final products, both of the two carbonate sources were added with amount of 1–12 mmol after the mineralizer fully dissolved. The hydrothermal treatment process was the same as that of pure hydroxyapatite synthetic procedure, and the products were washed and stored as before accordingly. The crystallization of HA and CHA followed the reaction equation as-shown in the following reactions:

(10 − (x/2))Ca(NO_3_)_2_ + (6 − (2/3)x)KH_2_PO_4_ + 14KOH + xK_2_CO_3_ → Ca_10−(x/2)_(PO_4_)_6−(2/3)x_(CO_3_)_x_(OH)_2_ + (20 + (4/3)x)K^+^ + (20 + (4/3)x)NO_3_^−^ + 12H_2_O (5)

#### 3.2.3. Synthesis of Rare-Earth Doped Hydroxyapatite and Carbonated-Hydroxyapatite Nanocrystals

Rare-earth doped samples were synthesized with a similar procedure as that of pure hydroxyapatite sample and carbonated hydroxyapatite samples with the addition of stoichiometric amounts of RE(NO_3_)_3_ solutions before KOH mineralizer. For simplicity, five representative RE^3+^ ions were selected as the model to assess the accommodative capability of carbonated hydroxyapatite, i.e., Pr^3+^, Sm^3+^, Eu^3+^, Tb^3+^, and Ho^3+^. The synthetic procedure for each dopant sample was kept the same with merely substituted RE^3+^ by adding the respective RE(NO_3_)_3_ solutions. White suspension particles formed in the beaker as soon as the addition of KOH. The whole mixture was vigorously stirred for 10 min before it was transferred into the autoclave. Various RE-doping level samples were synthesized with a same procedure by stoichiometrically control the atomic ratio of RE/(RE + Ca) with 1%, 2%, 3%, 4%, and 5%, respectively. The hydrothermal reaction was carried on at 200 °C for 24 h. The as-synthesized products were washed with deionized water for several times until the pH reaches 7–8. All of the samples were dried and kept in the same condition as that of HA.

### 3.3. Characterizations

Pourbaix (E-pH) diagrams for the calcium-carbonate-phosphate system were depicted based on the thermodynamic data provided by HSC Chemistry 6.0 software (Outo Kumpu Technology, Helsinki, Finland) at 37 °C and 200 °C, respectively. The pressure of the reactant system is defined as the saturated vapor pressure in the aqueous medium for each temperature, with the dielectric constant of the system of 74.182 and 34.925 at 37 and 200 °C, respectively. The starting molarity for each species of Ca, P, and C in any form of the compounds with a total molarity is 1.0 mol/kg. Sample morphology for all as-synthesized hydroxyapatite nanocrystals were measured with a JSM-7800F field-emission scanning electron microscope (SEM) (JEOL Ltd., Tokyo, Japan). Product composition was determined by inductively coupled plasma atomic emission spectroscopy (ICP-AES) and was also confirmed by energy dispersive spectroscopy (EDS). The EDS information has been collected for 60 s at 15 kV. For the measurement of ICP-AES, a mixed solution were prepared by dissolving 5–10 mg samples into 2 mL concentrated HCl and diluted to 50 mL volumetric flask. For EDS measurement, the characterized X-ray signals for Ca, RE, P, and O elements were collected in a 5 × 5 μm^2^ square region that was bombarded with 15 kV electrons for 60 s. The as-obtained spectra were analysed with elemental quantifying software from Oxford Company. Phase purity and crystal orientation for the self-assembled nanostructures were determined by powder X-ray diffraction (XRD) data, which were collected on a Rigaku D/Max 2500 V/PC X-ray diffractometer (Rigaku, Tokyo, Japan) with CuKα radiation (λ = 0.15406 nm) at 40 kV and 500 mA with a scan speed of 1 degree per minute at room temperature. The goniometer of the equipment was RINT2000 vertical goniometer with the samples loaded in a standard sample holder in a continuous scanning mode. The step scanning was in the angle range of 4° ≤ 2θ ≤ 120° with an increment of 0.02°. Rietveld refinement was performed with GSAS/EXPGUI full spectra fitting suite. Crystal structure for Ca_10_(PO_4_)_6_(OH)_2_ was depicted by a Diamond Software with ICSD card No. 26204 as a primary model. Fourier transform infrared (FTIR) spectra of various RE-doped HA nanocrystal samples were measured in the transmission mode by a Bruker Optics-IFS-66 V/S spectrometer in the wavenumber range of 400–4000 cm^−^^1^. The emission and excitation spectra of the phosphors were recorded using an Edinburgh Instruments FLS 920 spectrofluorometer equipped with a continuous 450 W xenon lamp and using a R928 photomultiplier tube detector. Lifetime measurements were performed using the same spectrophotometer and detectors using a 100 W pulsed xenon lamp mF920H (200–900 nm, 10–100 Hz). The bi-exponential decay curves were fitted with Origin^®^ 8.5 (OriginLab Corporation, Northampton, MA, USA).

### 3.4. Cell Culture and Vialability

Commercial HA (purity > 98%) powder was bought from Kunshan Chinese Technology New Materials Co., Ltd. MC3T3-E1 cells were cultured in Dulbecco’s modified eagle medium (DMEM), which contains 10% fetal bovine serum (FBS), 100 units/mL penicillin, 100 mg/mL streptomycin, in carbon dioxide incubator at 37 °C. The medium was updated every 3 days and culture for the generations of propagation when the filling capacity over 85% at the bottom of the bottle. The extract liquor of the powder product was made based on the ISO-10993-5 and ISO-10993-12 standard files. Various forms of apatites, i.e., hydroxyapatite, carbonated-hydroxyapatite, Eu^3+^- and Tb^3+^-substituted HA and CHA with an amount of 2 g were added in 10 mL DMEM and extracted for 24 h at 37 °C. Then, 10% of FBS, 100 units/mL of penicillin, 100 mg/mL of streptomycin were added. About 3000 cells for each 96-well-plates were inoculated for each well with three replicates. After inoculation, 100 μL DMEM was supplied for each well, which were replaced every day. Cell proliferation were recorded by a cell counting kit-8 (CCK-8, New Cell & Molecular Biotech Co., Ltd., Suzhou, China). The absorbance value were recorded by a microplate reader (Thermo Scientific, Waltham, MA, USA) at 450 nm for each well after the 10 μL CCK-8 solution addition for the 1st, 3rd, and 5th day, respectively. The cell proliferation levels were assessed via MTT method. The absorbance data of each group was quantified and averaged from three individual tests. Statistical data in each experiment were analyzed via SPSS 19.0 software (Armonk, NY, USA), with the notation of x ± s stands for the data and standard deviation. One-way analysis of variance analysis was applied to illustrate the statistical significance between the samples, which is marked by *, **, and *** for *p* < 0.05, *p* < 0.01, and *p* < 0.001, respectively.

## 4. Conclusions

In summary, based on the Pourbaix diagram of the thermodynamic data of calcium, phosphate, carbonate, and rare-earth, we have developed an efficient method to synthesize B-type carbonated hydroxyapatite nanocrystals with controlled C/P ratio and the rare-earth doped CHA materials. Temperature and environment pressure play impact on their existence partition thermodynamically via a balance factor, which are crucial for the mineralization of carbonate and phosphate simultaneously. Structural analysis reveals that the planar shape of carbonate anions substitute tetrahedral shape of phosphates by superimposed on one of the faces of PO_4_^3−^ unit with a 30° rotation in reference to c-axis. The introduction of carbonate in hydroxyapatite changes the shape of nanorod with the length-to-diameter ratio from 3.6 to 2.9 with increased crystal sizes. Type of rare-earth could play an important role on the crystal facet stabilization and shape formation. In vitro experiments for the bioactivity assay demonstrated that all of the synthesized (carbonated-)hydroxyapatite materials are nontoxic, with the cell viability slightly superior to that of commercial HA material. Our results have demonstrated that the carbonated hydroxyapatite with controllable carbonate ratio could meet the similar physiochemical characters as that of human bone [92]. Although the complete repair of demineralized dentin is still challenged by the systematic relationship of tissue scaffold, soluble matrix and minerals [93], the rare-earth labelled carbonated-hydroxyapatites with site-selectivity could provide a baseline for tracing the demineralization of natural biologically derived apatite (e.g., human tooth) in vivo [94]. The Pourbaix guided mechanism in the mineralization of carbonated hydroxyapatite is also useful the understanding the formation of concentration gradient composition of naturally mineralized hard tissue for the management of structural engineering of new biomedical materials.

## Figures and Tables

**Table 1:** Ionic radii of Ca^2+^ and RE^3+^ in RE doped hydroxyapatite with the coordinated number of VI and VII ^a^.

Cations	Radii of Cations in Coordinated Number	
	VI	VII
Ca^2+^	100 pm	106 pm
Pr^3+^	99 pm	- ^b^
Sm^3+^	95.8 pm	102 pm
Eu^3+^	94.7 pm	101 pm
Tb^3+^	92.3 pm	98 pm
Ho^3+^	90.1 pm	-

^a^ Ionic radii data were taken from Shannon in ref. [80]. ^b^ Sign of—is denoted as unfeasible in reference.

**Table 2:** Composition analysis of Ca^2+^ and Tb^3+^ in hydrothermally synthesized Tb-doped hydroxyapatite (HA) and CHA samples.

Sample	EDS Results	ICP-AES Results
0% Tb-HA	Ca:Tb = 1:0	Ca:Tb = 1:0.0001
1% Tb-HA	Ca:Tb = 1:0.0089	Ca:Tb = 1:0.0096
2% Tb-HA	Ca:Tb = 1:0.0166	Ca:Tb = 1:0.0182
3% Tb-HA	Ca:Tb = 1:0.0245	Ca:Tb = 1:0.0275
4% Tb-HA	Ca:Tb = 1:0.0312	Ca:Tb = 1:0.0367
5% Tb-HA	Ca:Tb = 1:0.0378	Ca:Tb = 1:0.0445
0% Tb-CHA	Ca:Tb = 1:0	Ca:Tb = 1:0.0000
1% Tb-CHA	Ca:Tb = 1:0.0093	Ca:Tb = 1:0.0096
2% Tb-CHA	Ca:Tb = 1:0.0176	Ca:Tb = 1:0.0185
3% Tb-CHA	Ca:Tb = 1:0.0261	Ca:Tb = 1:0.0280
4% Tb-CHA	Ca:Tb = 1:0.0331	Ca:Tb = 1:0.0373
5% Tb-CHA	Ca:Tb = 1:0.0389	Ca:Tb = 1:0.0452

## Data Availability

The data contained in the paper are available from the authors.

## Supplementary Materials

The following are available online. Figure S1: Pourbaix diagram of phosphate in aqueous at 37 °C. Figure S2: Pourbaix diagram of calcium in aqueous at 37 °C. Figure S3: Pourbaix diagram of the system of calcium and phosphate in aqueous at 37 °C. Figure S4: Pourbaix diagram of phosphate in aqueous at 200 °C. Figure S5: Pourbaix diagram of calcium in aqueous at 200 °C. Figure S6: Pourbaix diagram of the system of calcium and phosphate in aqueous at 200 °C. Figure S7: Pourbaix diagram of carbonate in aqueous at 37 °C. Figure S8: Pourbaix diagram of carbonate in aqueous at 200 °C. Figure S9: Pourbaix diagram of the system of calcium, carbonate and phosphate in aqueous at 37 °C. Figure S10: Pourbaix diagram of the system of calcium, carbonate and phosphate in aqueous at 200 °C. Figure S11: PXRD data and the Rietveld refinement results of CHA with varied amounts of K_2_CO_3_ for the starting reactant in the same hydrothermal synthetic process. Figure S12: Pourbaix diagram of Tb-species in aqueous at 37 °C. Figure S13: Pourbaix diagram of Tb-species in aqueous at 200 °C. Figure S14: SEM of (a) HA:5%Tb and (b) CHA:5%Tb samples. Figure S15: PXRD pattern of Pr-doped carbonated hydroxyapatite nanocrystals with increasing doping level from x = 0.01 to 0.08, respectively. Figure S16: PXRD pattern of Sm-doped carbonated hydroxyapatite nanocrystals with increasing doping level from x = 0.01 to 0.08, respectively. Figure S17: PXRD pattern of Ho-doped carbonated hydroxyapatite nanocrystals with increasing doping level from x = 0.01 to 0.08, respectively. Figure S18: FT-IR spectra of various (a) Sm^3+^ and (b) Ho^3+^ doped hydroxyapatite samples, respectively. Figure S19: SEM graphs of various (a) Sm^3+^ and (b) Ho^3+^ doped hydroxyapatite samples, respectively. Figure S20: EDS spectra of Tb^3+^-doped CHA samples with different Tb^3+^ atomic ratio. Figure S21: Excitation spectra of Tb^3+^ in CHA crystal lattice monitoring the emission at 452 nm. Figure S22: Emission spectra of Pr^3+^, Sm^3+^, Eu^3+^, and Ho^3+^ in CHA crystal lattice with the excitation wavelength at 350 nm. Figure S23: Decay times of (a) HA:1%Tb, (b) HA:2%Tb, (c) HA:3%Tb, (d) HA:4%Tb, (e) HA5%Tb, (f) CHA:1%Tb, (g) CHA:2%Tb, (h) CHA:3%Tb, (i) CHA:4%Tb, (j) CHA:5%Tb, respectively. Table S1: Observed vibrational frequencies and band assignments of carbonate-substituted hydroxyapatite samples. Table S2: Rietveld fitting results of CHA with different carbonate ratio. Table S3: Atomic sites in carbonated hydroxyapatite synthesized via hydrothermal method with K_2_CO_3_ as the sole carbonate source. Table S4: Calculated crystallite size and measured average size of CHA nanocrystals. Table S5: PL peak position and the fitted results for the decay curve of each apatite samples.

## Sample Availability

Samples of the compounds are not available from the authors.
