# Supplementary material for: Pourbaix-Guided Mineralization and Site-Selective Photoluminescence Properties of Rare Earth Substituted B-Type Carbonated Hydroxyapatite Nanocrystals
Source: Molecules. 2021 Jan 21;26(3):540. doi: 10.3390/molecules26030540 (PMC7864488; doi:10.3390/molecules26030540)
Supplement: Supplementary file 1 [file molecules-26-00540-s001.pdf]

# Electronic Supplementary Materials

# Pourbaix-Guided Mineralization and Site-Selective Photoluminescence Properties of Rare Earth Substituted B-Type Carbonated Hydroxyapatite Nanocrystals

**Peng Liu [1], Zhengqiang Li [1], Long Yuan [2,*], Xiaolin Sun [1] and Yanmin Zhou [1,*]**

[1] School of Stomatology, Jilin University, Changchun 130021, China; pengliu18@mails.jlu.edu.cn (P.L.); zqli13@mails.jlu.edu.cn (Z.L.); sunxiaolin@jlu.edu.cn (X.S.)
[2] Key Laboratory of Functional Materials Physics and Chemistry of the Ministry of Education, College of Physics, Jilin Normal University, Changchun 130103, China
* Correspondence: yuanlong@jlnu.edu.cn (L.Y.); zhouym@jlu.edu.cn (Y.Z.)

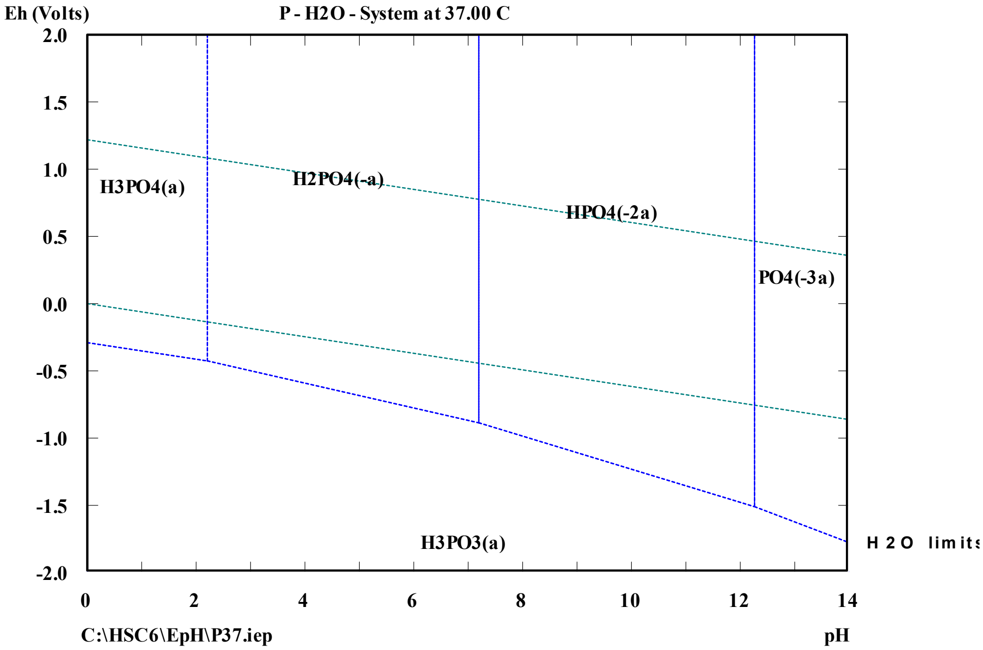


| ELEMENTS | Molality | Pressure |
|---|---|---|
| P | 1.000E+00 | 1.000E+00 |

**Figure S1.** Pourbaix diagram of phosphate in aqueous at 37 °C. For the Pourbaix analysis, phosphate species of $H_3PO_4$, $H_3PO_3$(a), $H_3PO_4$(a), $HPO_4^{2-}$, $H_2PO_4^{-}$ and $PO_4^{3-}$ were accounted with the enthalpy of formation of −265.887, −203.734, −273.318, −259.976, −270.186, −242.575 kcal/mol for each species, respectively.

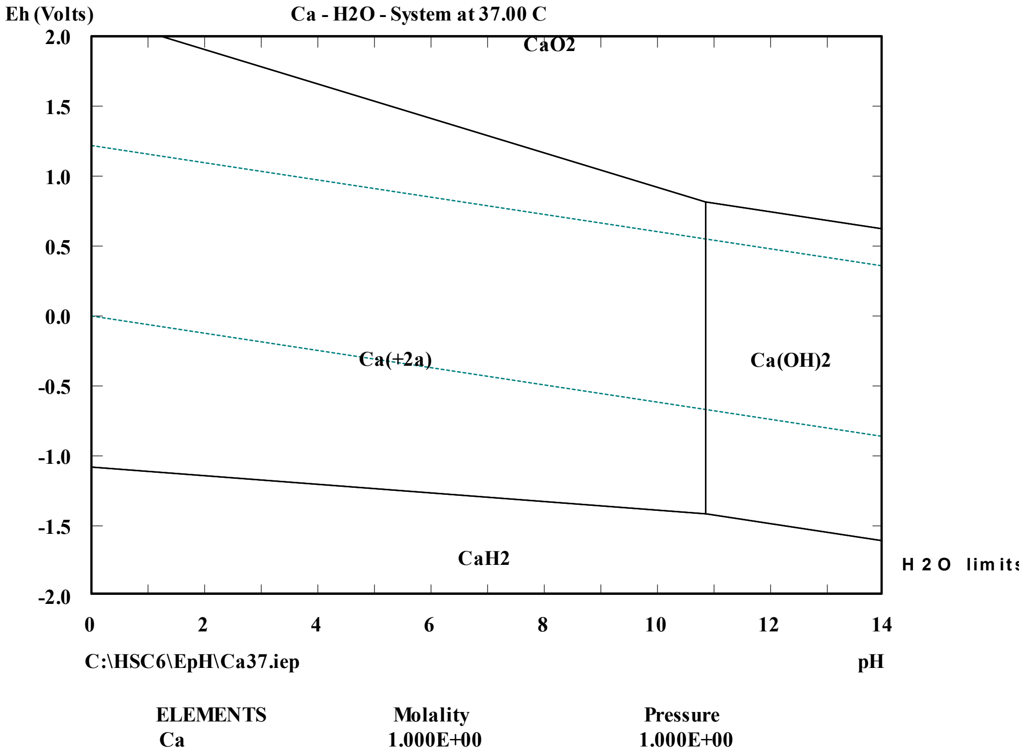


**Figure S2.** Pourbaix diagram of calcium in aqueous at 37 °C. For the Pourbaix analysis, calcium species of Ca, $CaH_2$, CaO, $CaO_2$, $Ca(OH)_2$, $Ca^{2+}$ and $CaOH^+$ were accounted with the enthalpy of formation of 0, −32.598, −143.887, −145.419, −213.841, −132.216, −170.990 kcal/mol for each species, respectively.

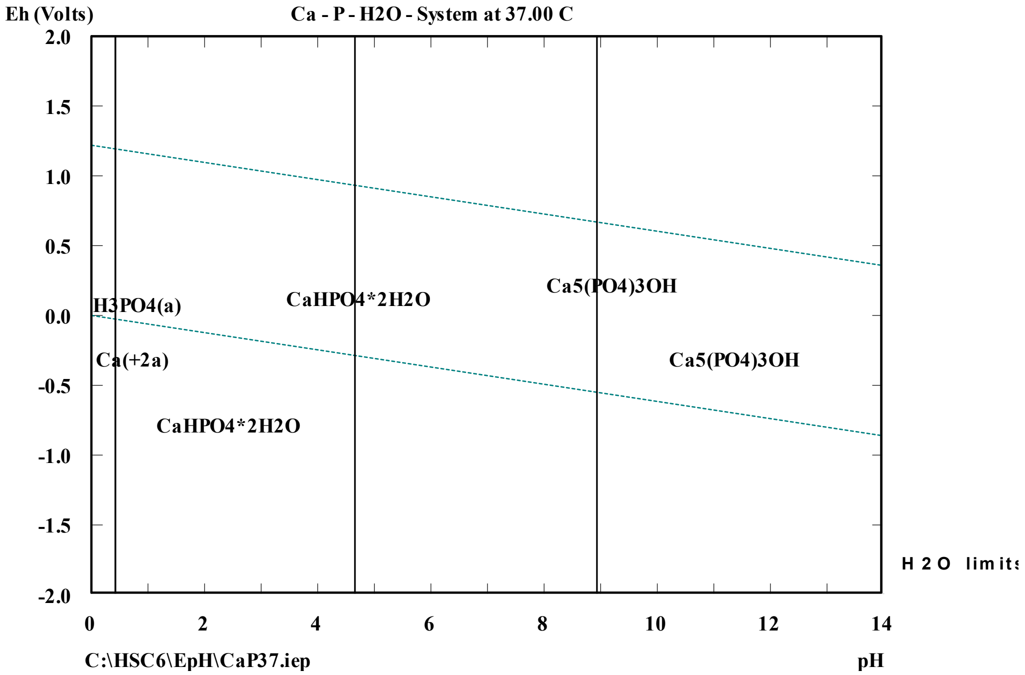


| ELEMENTS | Molality | Pressure |
|---|---|---|
| Ca | 1.000E+00 | 1.000E+00 |
| P | 1.000E+00 | 1.000E+00 |

**Figure S3.** Pourbaix diagram of the system of calcium and phosphate in aqueous at 37 °C. For the Pourbaix analysis, calcium species of $CaHPO_4$, $Ca(H_2PO_4)_2$, $CaHPO_4.2H_2O$, $Ca(H_2PO_4)_2.H_2O$, $Ca(OH)_2$, $Ca_3(PO_4)_2$, $Ca_5(PO_4)_3(OH)$, $Ca^{2+}$, $CaOH^+$, $H_3PO_4$, $HPO_4^{2-}$, $H_2PO_4^-$ and $PO_4^{3-}$ were accounted with the enthalpy of formation of −400.518, −666.713, −516.754, −730.590, −213.841, −926.218, −1507.194, −132.216, −170.990, −273.318, −259.976, −270.186, 242.575 kcal/mol for each species, respectively.

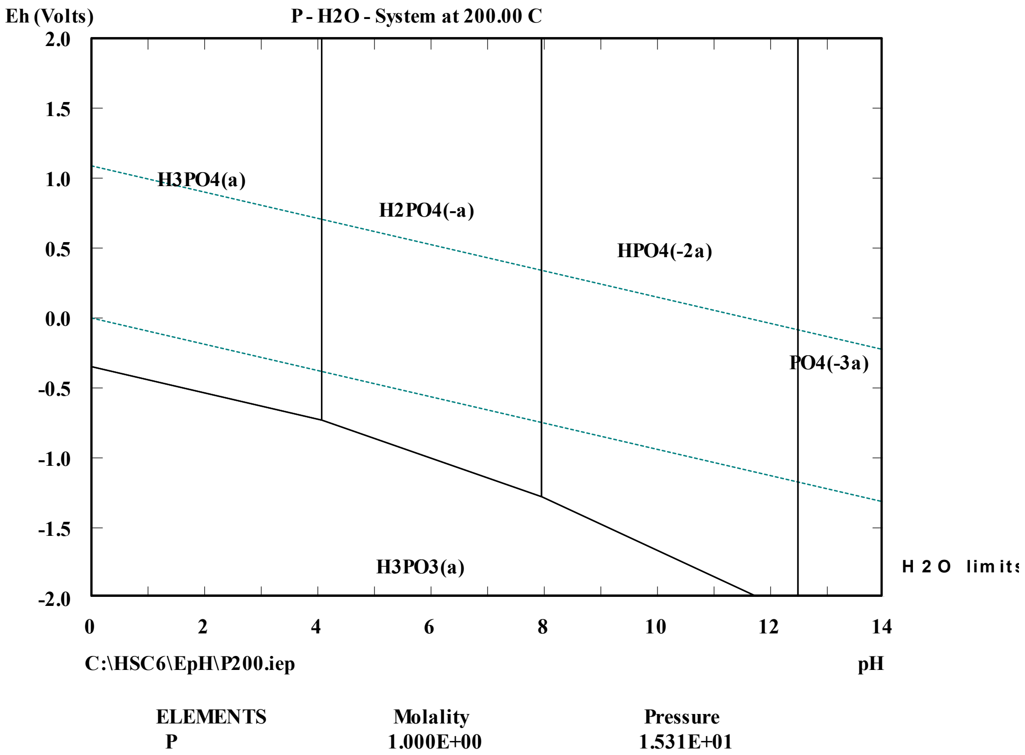


**Figure S4.** Pourbaix diagram of phosphate in aqueous at 200 °C. For the Pourbaix analysis, phosphate species of $H_3PO_4$, $H_3PO_3$(a), $H_3PO_4$(a), $HPO_4^{2-}$, $H_2PO_4^-$ and $PO_4^{3-}$ were accounted with the enthalpy of formation of −246.745, −189.408, −255.629, −229.607, −246.823, −202.581 kcal/mol for each species, respectively.

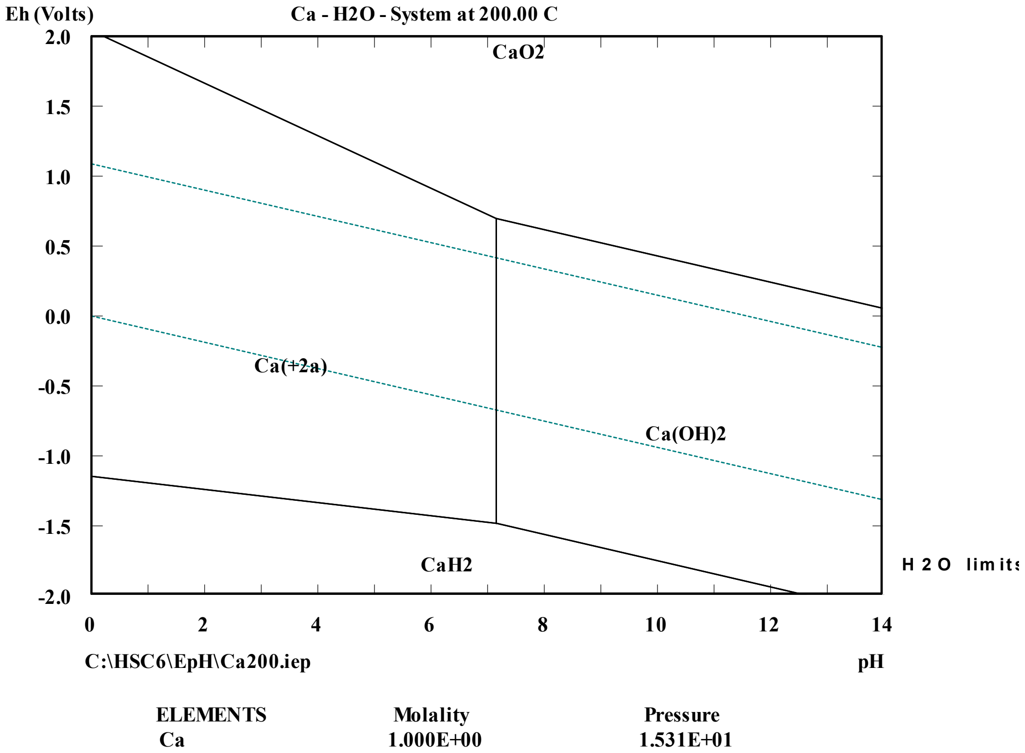


**Figure S5.** Pourbaix diagram of calcium in aqueous at 200 °C. For the Pourbaix analysis, calcium species of Ca, $CaH_2$, CaO, $CaO_2$, $Ca(OH)_2$, $Ca^{2+}$ and $CaOH^{+}$ were accounted with the enthalpy of formation of 0, −27.374, −139.794, −139.344, −202.463, −133.066, −166.149 kcal/mol for each species, respectively.

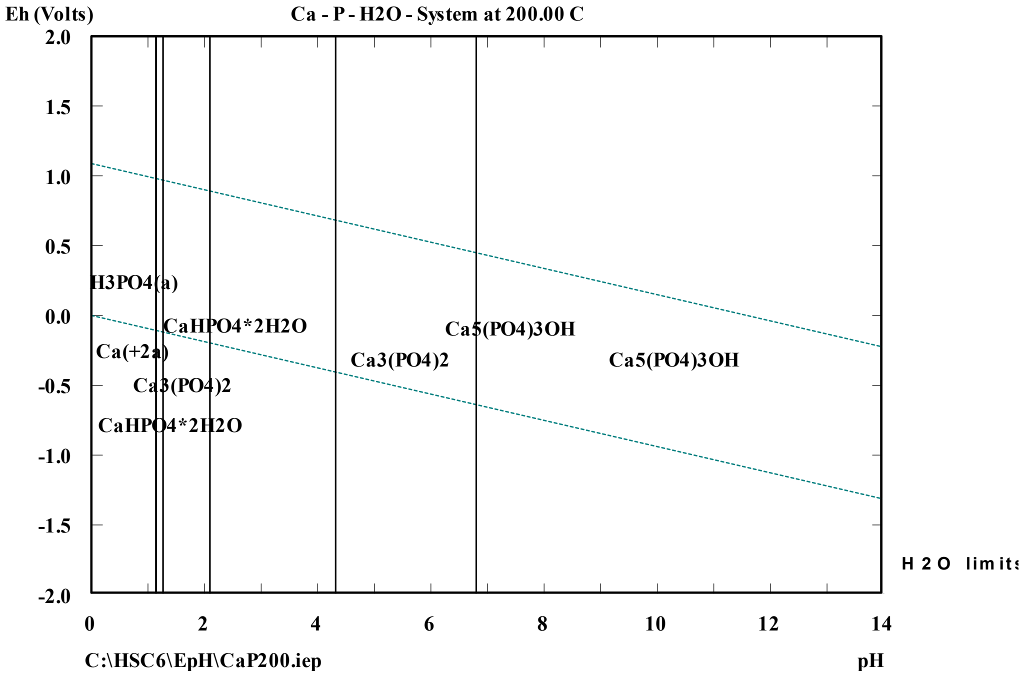


| ELEMENTS | Molality | Pressure |
|---|---|---|
| Ca | 1.000E+00 | 1.531E+01 |
| P | 1.000E+00 | 1.531E+01 |

**Figure S6.** Pourbaix diagram of the system of calcium and phosphate in aqueous at 200 °C. For the Pourbaix analysis, calcium species of $CaHPO_4$, $Ca(H_2PO_4)_2$, $CaHPO_4.2H_2O$, $Ca(H_2PO_4)_2.H_2O$, $Ca(OH)_2$, $Ca_3(PO_4)_2$, $Ca_5(PO_4)_3(OH)$, $Ca^{2+}$, $CaOH^+$, $H_3PO_4$, $HPO_4^{2-}$, $H_2PO_4^-$ and $PO_4^{3-}$ were accounted with the enthalpy of formation of −383.009, −627.024, −484.116, −684.089, −202.463, −895.083, −1454.806, −133.066, −166.149, −255.629, −229.607, −246.823, 202.581 kcal/mol for each species, respectively.

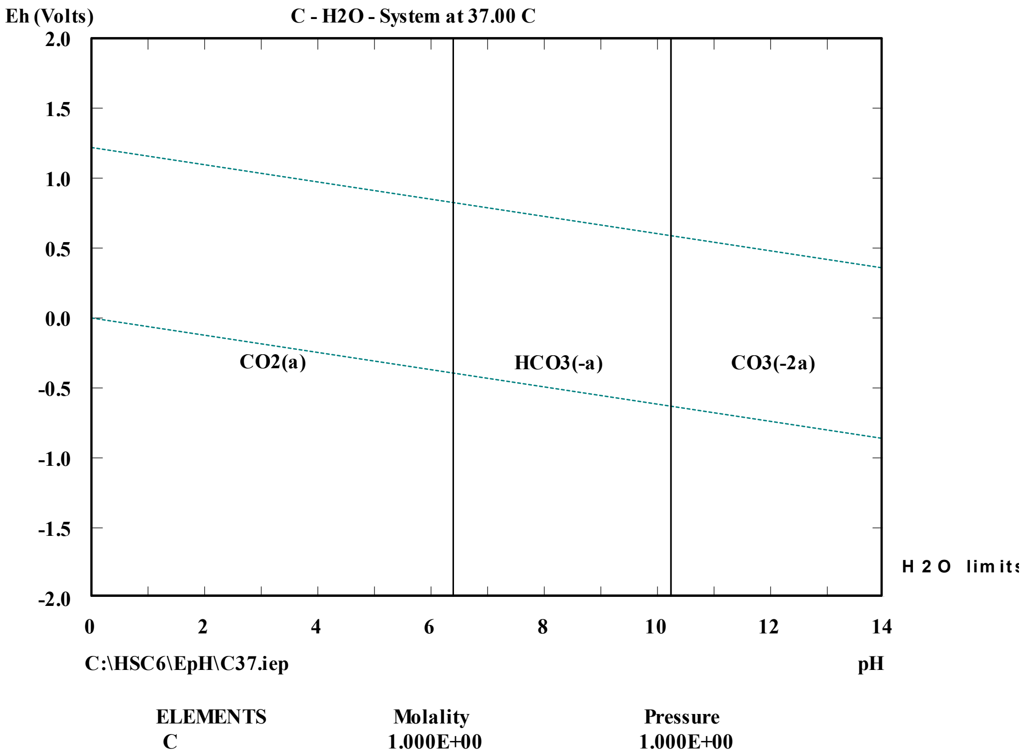


**Figure S7.** Pourbaix diagram of carbonate in aqueous at 37 °C. For the Pourbaix analysis, carbonate species of $CO_2$(a), $CO_3^{2-}$, $H_2CO_3$(a), and $HCO_3^-$ were accounted with the enthalpy of formation of −92.124, −124.375, −148.207, −139.266 kcal/mol for each species, respectively.

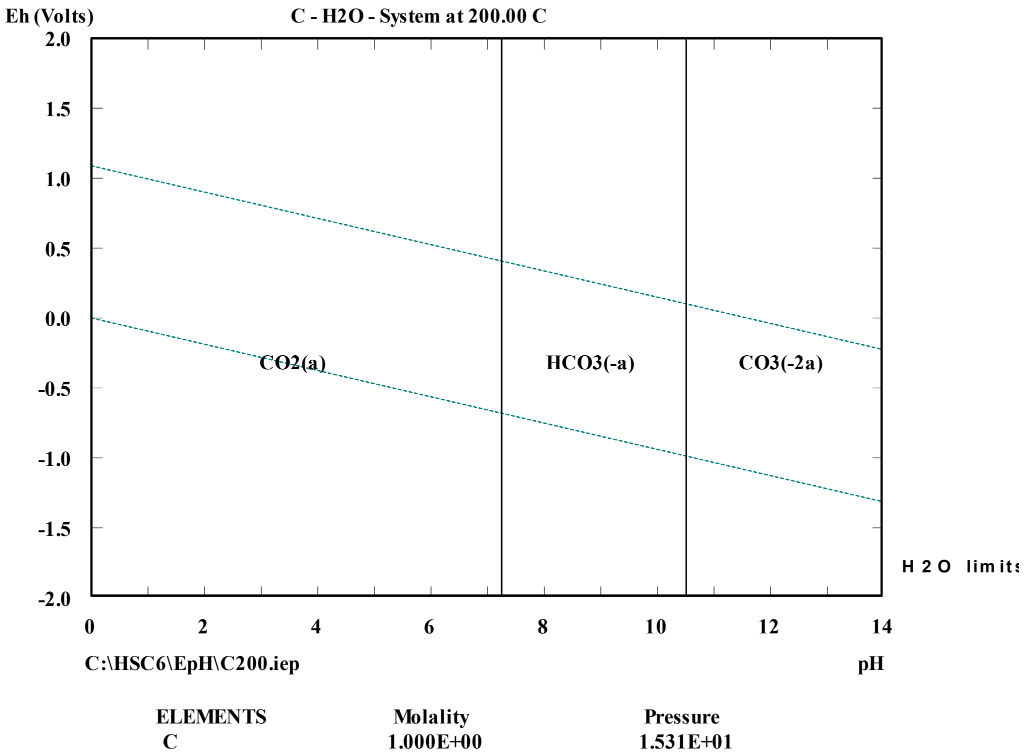


**Figure S8.** Pourbaix diagram of carbonate in aqueous at 200 °C. For the Pourbaix analysis, carbonate species of $CO_2$(a), $CO_3^{2-}$, $H_2CO_3$(a), and $HCO_3^-$ were accounted with the enthalpy of formation of −90.058, −101.802, −137.340, −124.552 kcal/mol for each species, respectively.

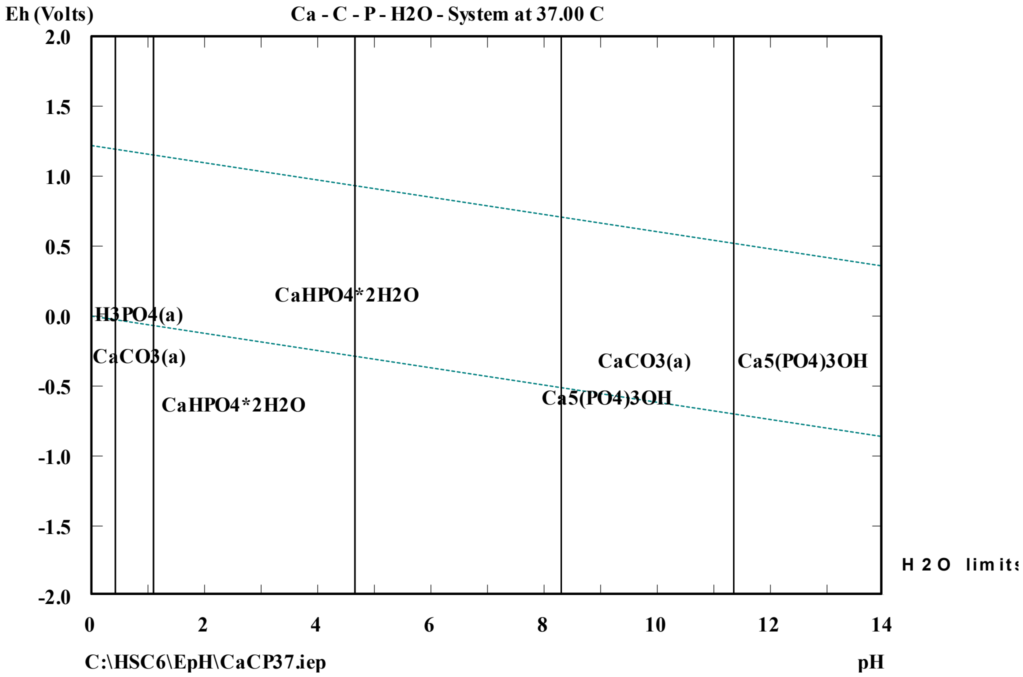


| ELEMENTS | Molality | Pressure |
|---|---|---|
| Ca | 1.000E+00 | 1.000E+00 |
| C | 1.000E+00 | 1.000E+00 |
| P | 1.000E+00 | 1.000E+00 |

**Figure S9.** Pourbaix diagram of the system of calcium, carbonate and phosphate in aqueous at 37 °C. For the Pourbaix analysis, calcium species of $CaCO_3$, $CaHPO_4$, $Ca(H_2PO_4)_2$, Ca-$HPO_4.2H_2O$, $Ca(H_2PO_4)_2.H_2O$, $Ca(OH)_2$, $Ca_3(PO_4)_2$, $Ca_5(PO_4)_3(OH)$, $CO_3^{2-}$, $Ca^{2+}$, $CaCO_3$, $HCO_3^-$, $H_3PO_4$, $HPO_4^{2-}$, $H_2PO_4^-$ and $PO_4^{3-}$ were accounted with the enthalpy of formation of −268.864, −400.518, −666.713, −516.754, −730.590, −213.841, −926.218, −1507.194, −124.735, −132.216, −271.835, −139.266, 273.318, 259.976, −270.186, −242.575 kcal/mol for each species, respectively.

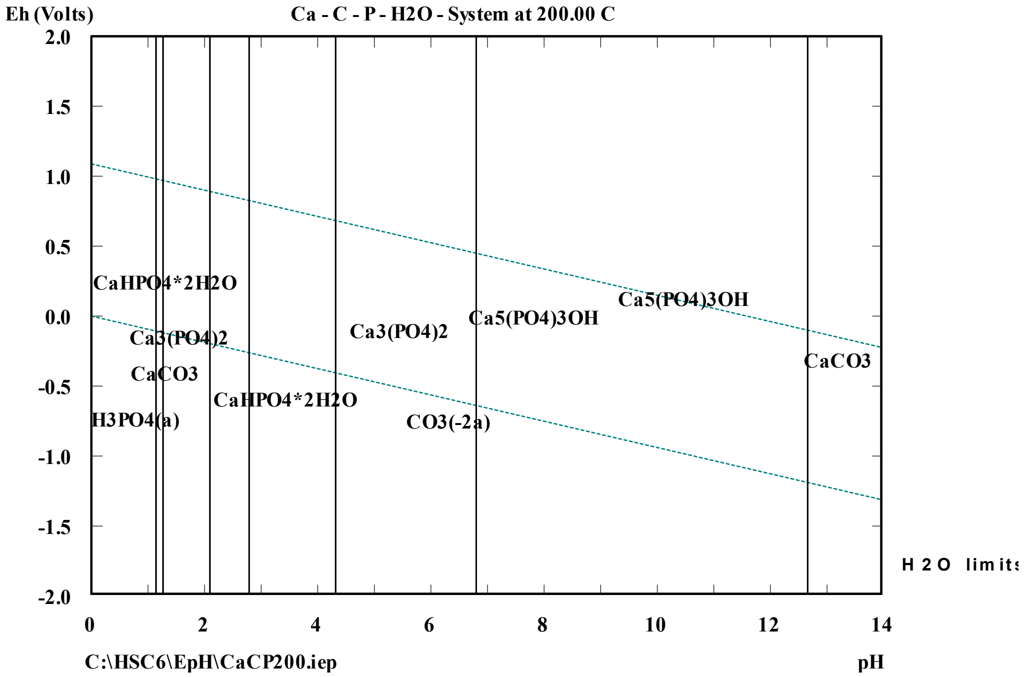


| ELEMENTS | Molality | Pressure |
|---|---|---|
| Ca | 1.000E+00 | 1.531E+01 |
| C | 1.000E+00 | 1.531E+01 |
| P | 1.000E+00 | 1.531E+01 |

**Figure S10.** Pourbaix diagram of the system of calcium, carbonate and phosphate in aqueous at 200 °C. For the Pourbaix analysis, calcium species of $CaCO_3$, $CaHPO_4$, $Ca(H_2PO_4)_2$, $CaHPO_4.2H_2O$, $Ca(H_2PO_4)_2.H_2O$, $Ca(OH)_2$, $Ca_3(PO_4)_2$, $Ca_5(PO_4)_3(OH)$, $CO_3^{2-}$, $Ca^{2+}$, $CaCO_3$, $HCO_3^-$, $H_3PO_4$, $HPO_4^{2-}$, $H_2PO_4^-$ and $PO_4^{3-}$ were accounted with the enthalpy of formation of −258.707, −383.009, −627.024, −484.116, −684.089, −202.463, −895.083, −1454.806, −101.802, −133.066, −256.758, −124.552, 255.629, 229.607, −246.823, −202.581 kcal/mol for each species, respectively. Chemical formulas on the lines indicate that this phase is stable in both of the two neighbor regions.

**Table S1.** Observed vibrational frequencies and band assignments of carbonate-substituted hydroxyapatite samples.

| Vibration | CHA1 | CHA2 | CHA3 | CHA4 | CHA5 | CHA6 | CHA7 | CHA8 | CHA9 | CHA10 | CHA11 | CHA12 |
|---|---|---|---|---|---|---|---|---|---|---|---|---|
| Out-of-plane bending O-P-O in $PO_4^{3-}$ | 410 | 409 | 410 | 408 | 409 | 409 | 410 | 408 | 409 | 409 | 410 | 408 |
| In-plane bending $PO_4^{3-}$ bend $\nu_4$ | 565.5 | 564.1 | 564.1 | 564.1 | 562.7 | 564.1 | 562.7 | 564.1 | 562.7 | 564.1 | 562.7 | 564.1 |
| $PO_4^{3-}$ bend $\nu_4$ | 602.7 | 602.7 | 602.7 | 602.7 | 602.7 | 602.7 | 602.7 | 602.7 | 602.7 | 602.7 | 602.7 | 602.7 |
| $[OH]^-$ librational mode | 632.6 | 632.7 | 632.7 | 632.7 | 632.7 | 632.7 | 632.7 | 632.7 | 632.7 | 632.7 | 632.7 | 632.7 |
| Out-of-phase bending of $CO_3^{2-}$ | 875.4 | 875.4 | 875.4 | 875.4 | 875.4 | 875.4 | 875.4 | 875.4 | 875.4 | 875.4 | 875.4 | 875.4 |
| $PO_4^{3-}$ stretch $\nu_1$ | 962.5 | 962.5 | 962.5 | 962.5 | 962.5 | 962.5 | 962.5 | 962.5 | 962.5 | 962.5 | 962.5 | 962.5 |
| $PO_4^{3-}$ stretch $\nu_3$ | 1012-1118 | 1019-1108 | 1031-1092 | 1021-1091 | 1021-1112 | 1021-1107 | 1021-1052 | 1021-1111 | 1021-1112 | 1023-1105 | 1021-1052 | 1021-1091 |
| $CO_3^{2-}$ stretching vibrations | 1385.3 | 1385.3 | 1385.3 | 1385.3 | 1385.3 | 1385.3 | 1385.3 | 1385.3 | 1385.3 | 1385.3 | 1385.3 | 1385.3 |
| Stretching of $CO_3^{2-}$ | 1421,1469.5 | 1421,1469.5 | 1421,1471 | 1421,1470 | 1421,1469.5 | 1421,1469.5 | 1421,1471 | 1421,1470 | 1421,1469.5 | 1421,1469.5 | 1421,1471 | 1421,1470 |
| In-plane bending of H-O-H | 1638 | 1646.6 | 1646.6 | 1646.6 | 1646.6 | 1646.6 | 1646.6 | 1646.6 | 1646.6 | 1646.6 | 1646.6 | 1646.6 |
| $[OH]^-$ stretching mode | 3571.7 | 3571.7 | 3571.7 | 3571.7 | 3571.7 | 3571.7 | 3571.7 | 3571.7 | 3571.7 | 3571.7 | 3571.7 | 3571.7 |

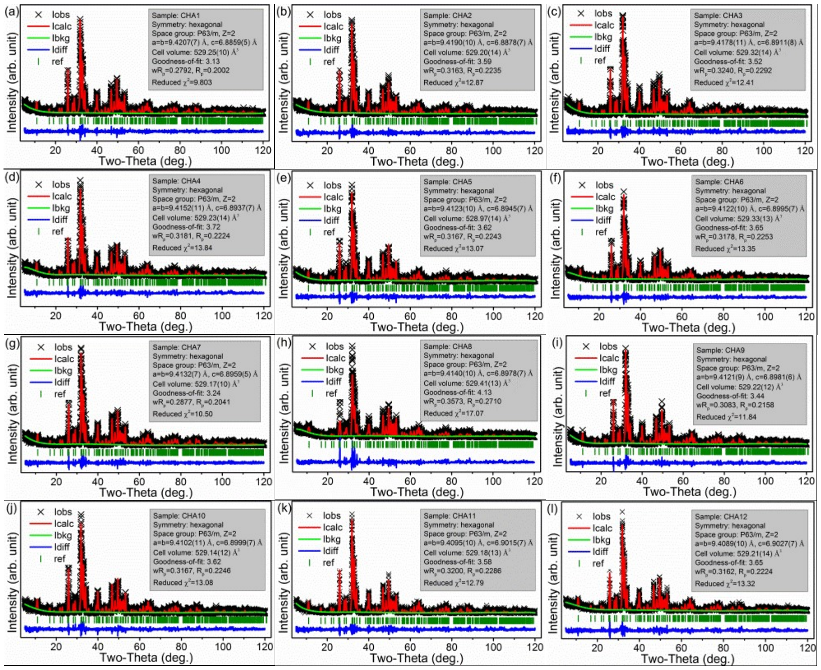


**Figure S11.** PXRD data and the Rietveld refinement results of CHA with varied amounts of $K_2CO_3$ for the starting reactant in the same hydrothermal synthetic process: (**a**) 1.0 mmol (0.1382 g), (**b**) 2.0 mmol (0.2764 g), (**c**) 3.0 mmol (0.4146 g), (**d**) 4.0 mmol (0.5528 g), (**e**) 5.0 mmol (0.6910 g), (**f**) 6.0 mmol (0.8292 g), (**g**) 7.0 mmol (0.9674 g), (**h**) 8.0 mmol (1.1056 g), (**i**) 9.0 mmol (1.2438 g), (**j**) 10.0 mmol (1.3820 g), (**k**) 11.0 mmol (1.5202 g), (**l**) 12.0 mmol (1.6584 g), respectively. The notation of (X) is the observed X-ray intensity at each diffraction angle, and red, green, and blue lines indicate the theoretically fitted, background, and intensity differences between the observed and calculate results. Olive bars indicate the calculated diffraction peak positions for each PXRD data. Inset of the figures displayed the crystallographic information and refinement results for each CHA sample.

**Table S2.** Rietveld fitting results of CHA with different carbonate ratio.

| **formula** | **CHA1** | **CHA2** | **CHA3** | **CHA4** | **CHA5** | **CHA6** |
|---|---|---|---|---|---|---|
| symmetry | hexagonal | hexagonal | hexagonal | hexagonal | hexagonal | hexagonal |
| space group | P63/m | P63/m | P63/m | P63/m | P63/m | P63/m |
| a =b (Å) | 9.4207(7) | 9.4190(10) | 9.4178(11) | 9.4152(11) | 9.4123(10) | 9.4122(10) |
| c (Å) | 6.8859(5) | 6.8878(7) | 6.8911(8) | 6.8937(7) | 6.8945(7) | 6.8995(7) |
| $\alpha$=β (deg) | 90 | 90 | 90 | 90 | 90 | 90 |
| γ (deg) | 120 | 120 | 120 | 120 | 120 | 120 |
| Z | 2 | 2 | 2 | 2 | 2 | 2 |
| V (Å$^3$) | 529.25(10) | 529.20(14) | 529.32(14) | 529.23(14) | 528.97(14) | 529.33(13) |
| $R_p$ | 0.2002 | 0.2235 | 0.2292 | 0.2224 | 0.2243 | 0.2253 |
| $R_{wp}$ | 0.2792 | 0.3163 | 0.3240 | 0.3181 | 0.3167 | 0.3187 |
| $\chi^2$ | 9.803 | 12.87 | 12.41 | 13.84 | 13.07 | 13.35 |
| formula | CHA7 | CHA8 | CHA9 | CHA10 | CHA11 | CHA12 |
| symmetry | hexagonal | hexagonal | hexagonal | hexagonal | hexagonal | hexagonal |
| space group | P63/m | P63/m | P63/m | P63/m | P63/m | P63/m |
| a=b (Å) | 9.4132(7) | 9.4140(10) | 9.4121(9) | 9.4102(11) | 9.4095(10) | 9.4089(10) |
| c (Å) | 6.8959(5) | 6.8978(7) | 6.8981(6) | 6.8999(7) | 6.9015(7) | 6.9027(7) |
| $\alpha$=β (deg) | 90 | 90 | 90 | 90 | 90 | 90 |
| γ (deg) | 120 | 120 | 120 | 120 | 120 | 120 |
| Z | 2 | 2 | 2 | 2 | 2 | 2 |
| V (Å$^3$) | 529.17(10) | 529.41(13) | 529.22(12) | 529.14(12) | 529.18(13) | 529.21(14) |
| $R_p$ | 0.2041 | 0.2710 | 0.2158 | 0.2246 | 0.2286 | 0.2224 |
| $R_{wp}$ | 0.2877 | 0.3573 | 0.3083 | 0.3167 | 0.3200 | 0.3162 |
| $\chi^2$ | 10.50 | 17.07 | 11.84 | 13.08 | 12.79 | 13.32 |

**Table S3.** Atomic sites in carbonated hydroxyapatite synthesized via hydrothermal method with $K_2CO_3$ as the sole carbonate source.

| | atom | site | x | y | z | occupancy | $U_{iso}$ |
|---|---|---|---|---|---|---|---|
| CHA1 | Ca1 | 4f | 0.3333 | 0.6667 | −0.0037 | 1 | 0.0228 |
| | Ca2 | 6h | 0.2426 | 0.9870 | 0.25 | 1 | 0.0164 |
| | P1 | 6h | 0.3942 | 0.3692 | 0.25 | 0.955(5) | 0.0052 |
| | C1 | 6h | 0.3942 | 0.3692 | 0.25 | 0.045(5) | 0.0052 |
| | O1 | 6h | 0.3286 | 0.4697 | 0.25 | 1 | 0.0492 |
| | O2 | 6h | 0.5736 | 0.4541 | 0.25 | 1 | 0.0295 |
| | O3 | 12i | 0.3361 | 0.2568 | 0.0715 | 0.8 | 0.0055 |
| | O4 | 4e | 0 | 0 | 0.1728 | 0.5 | 0.0423 |
| CHA2 | atom | site | x | y | z | occupancy | $U_{iso}$ |
| | Ca1 | 4f | 0.3333 | 0.6667 | −0.0051 | 1 | 0.0236 |
| | Ca2 | 6h | 0.2442 | 0.9901 | 0.25 | 1 | 0.0203 |
| | P1 | 6h | 0.3930 | 0.3676 | 0.25 | 0.942(3) | 0.0065 |
| | C1 | 6h | 0.3930 | 0.3676 | 0.25 | 0.058(3) | 0.0065 |
| | O1 | 6h | 0.3302 | 0.4693 | 0.25 | 1 | 0.0336 |
| | O2 | 6h | 0.5785 | 0.4595 | 0.25 | 1 | 0.0360 |
| | O3 | 12i | 0.3373 | 0.2570 | 0.0698 | 0.8 | 0.0064 |
| | O4 | 4e | 0 | 0 | 0.1767 | 0.5 | 0.0026 |
| CHA3 | atom | site | x | y | z | occupancy | $U_{iso}$ |
| | Ca1 | 4f | 0.3333 | 0.6667 | −0.0033 | 1 | 0.0280 |
| | Ca2 | 6h | 0.2439 | 0.9886 | 0.25 | 1 | 0.0243 |
| | P1 | 6h | 0.3930 | 0.3665 | 0.25 | 0.934(14) | 0.0040 |
| | C1 | 6h | 0.3930 | 0.3665 | 0.25 | 0.066(14) | 0.0040 |
| | O1 | 6h | 0.3340 | 0.4694 | 0.25 | 1 | 0.0299 |
| | O2 | 6h | 0.5772 | 0.4622 | 0.25 | 1 | 0.0451 |
| | O3 | 12i | 0.3377 | 0.2584 | 0.0686 | 0.8 | 0.0055 |
| | O4 | 4e | 0 | 0 | 0.1865 | 0.5 | 0.0024 |
| CHA4 | atom | site | x | y | z | occupancy | $U_{iso}$ |
| | Ca1 | 4f | 0.3333 | 0.6667 | −0.0038 | 1 | 0.0264 |
| | Ca2 | 6h | 0.2441 | 0.9894 | 0.25 | 1 | 0.0163 |
| | P1 | 6h | 0.3919 | 0.3668 | 0.25 | 0.926(11) | 0.0012 |
| | C1 | 6h | 0.3919 | 0.3668 | 0.25 | 0.074(11) | 0.0012 |
| | O1 | 6h | 0.3345 | 0.4719 | 0.25 | 1 | 0.0286 |
| | O2 | 6h | 0.5784 | 0.4607 | 0.25 | 1 | 0.0507 |
| | O3 | 12i | 0.3375 | 0.2573 | 0.0702 | 0.8 | 0.0177 |
| | O4 | 4e | 0 | 0 | 0.1839 | 0.5 | 0.0456 |
| CHA5 | atom | site | x | y | z | occupancy | $U_{iso}$ |
| | Ca1 | 4f | 0.3333 | 0.6667 | −0.0074 | 1 | 0.0213 |
| | Ca2 | 6h | 0.2421 | 0.9886 | 0.25 | 1 | 0.0183 |
| | P1 | 6h | 0.3927 | 0.3678 | 0.25 | 0.909(7) | 0.0081 |
| | C1 | 6h | 0.3927 | 0.3678 | 0.25 | 0.091(7) | 0.0081 |

| | | | | | | | |
|---|---|---|---|---|---|---|---|
| | O1 | 6h | 0.3318 | 0.4674 | 0.25 | 1 | 0.0269 |
| | O2 | 6h | 0.5789 | 0.4596 | 0.25 | 1 | 0.0340 |
| | O3 | 12i | 0.3343 | 0.2576 | 0.0707 | 0.8 | 0.0119 |
| | O4 | 4e | 0 | 0 | 0.1782 | 0.5 | 0.0120 |
| CHA6 | atom | site | x | y | z | occupancy | $U_{iso}$ |
| | Ca1 | 4f | 0.3333 | 0.6667 | −0.0019 | 1 | 0.0273 |
| | Ca2 | 6h | 0.2452 | 0.9911 | 0.25 | 1 | 0.0239 |
| | P1 | 6h | 0.3927 | 0.3678 | 0.25 | 0.893(4) | 0.0075 |
| | C1 | 6h | 0.3927 | 0.3678 | 0.25 | 0.107(4) | 0.0075 |
| | O1 | 6h | 0.3323 | 0.4679 | 0.25 | 1 | 0.0359 |
| | O2 | 6h | 0.5767 | 0.4578 | 0.25 | 1 | 0.0330 |
| | O3 | 12i | 0.3351 | 0.2571 | 0.0707 | 0.8 | 0.0131 |
| | O4 | 4e | 0 | 0 | 0.1848 | 0.5 | 0.0183 |
| CHA7 | atom | site | x | y | z | occupancy | $U_{iso}$ |
| | Ca1 | 4f | 0.3333 | 0.6667 | −0.0062 | 1 | 0.0224 |
| | Ca2 | 6h | 0.2420 | 0.9874 | 0.25 | 1 | 0.0245 |
| | P1 | 6h | 0.3910 | 0.3666 | 0.25 | 0.884(6) | 0.0049 |
| | C1 | 6h | 0.3910 | 0.3666 | 0.25 | 0.116(6) | 0.0049 |
| | O1 | 6h | 0.3340 | 0.4730 | 0.25 | 1 | 0.0389 |
| | O2 | 6h | 0.5747 | 0.4575 | 0.25 | 1 | 0.0449 |
| | O3 | 12i | 0.3368 | 0.2563 | 0.0731 | 0.8 | 0.0083 |
| | O4 | 4e | 0 | 0 | 0.1764 | 0.5 | 0.0242 |
| CHA8 | atom | site | x | y | z | occupancy | $U_{iso}$ |
| | Ca1 | 4f | 0.3333 | 0.6667 | −0.0039 | 1 | 0.0210 |
| | Ca2 | 6h | 0.2448 | 0.9900 | 0.25 | 1 | 0.0152 |
| | P1 | 6h | 0.3948 | 0.3699 | 0.25 | 0.866(9) | 0.0069 |
| | C1 | 6h | 0.3948 | 0.3699 | 0.25 | 0.134(9) | 0.0069 |
| | O1 | 6h | 0.3379 | 0.4728 | 0.25 | 1 | 0.0290 |
| | O2 | 6h | 0.5765 | 0.4585 | 0.25 | 1 | 0.0436 |
| | O3 | 12i | 0.3380 | 0.2582 | 0.0617 | 0.8 | 0.0199 |
| | O4 | 4e | 0 | 0 | 0.1817 | 0.5 | 0.0956 |
| CHA9 | atom | site | x | y | z | occupancy | $U_{iso}$ |
| | Ca1 | 4f | 0.3333 | 0.6667 | −0.0053 | 1 | 0.0254 |
| | Ca2 | 6h | 0.2439 | 0.9897 | 0.25 | 1 | 0.0136 |
| | P1 | 6h | 0.3920 | 0.3689 | 0.25 | 0.844(8) | 0.0062 |
| | C1 | 6h | 0.3920 | 0.3689 | 0.25 | 0.156(8) | 0.0062 |
| | O1 | 6h | 0.3312 | 0.4686 | 0.25 | 1 | 0.0230 |
| | O2 | 6h | 0.5795 | 0.4613 | 0.25 | 1 | 0.0497 |
| | O3 | 12i | 0.3367 | 0.2578 | 0.0693 | 0.8 | 0.0150 |
| | O4 | 4e | 0 | 0 | 0.1791 | 0.5 | 0.0520 |
| CHA10 | atom | site | x | y | z | occupancy | $U_{iso}$ |
| | Ca1 | 4f | 0.3333 | 0.6667 | −0.0057 | 1 | 0.0172 |
| | Ca2 | 6h | 0.2431 | 0.9895 | 0.25 | 1 | 0.0173 |
| | P1 | 6h | 0.3923 | 0.3656 | 0.25 | 0.829(12) | 0.0175 |

| | | | | | | | |
|---|---|---|---|---|---|---|---|
| | C1 | 6h | 0.3923 | 0.3656 | 0.25 | 0.171(12) | 0.0175 |
| | O1 | 6h | 0.3313 | 0.4769 | 0.25 | 1 | 0.0559 |
| | O2 | 6h | 0.5816 | 0.4628 | 0.25 | 1 | 0.0779 |
| | O3 | 12i | 0.3367 | 0.2533 | 0.0638 | 0.8 | 0.0409 |
| | O4 | 4e | 0 | 0 | 0.1858 | 0.5 | 0.0833 |
| CHA11 | atom | site | x | y | z | occupancy | $U_{iso}$ |
| | Ca1 | 4f | 0.3333 | 0.6667 | −0.0056 | 1 | 0.0237 |
| | Ca2 | 6h | 0.2430 | 0.9894 | 0.25 | 1 | 0.0244 |
| | P1 | 6h | 0.3922 | 0.3669 | 0.25 | 0.818(5) | 0.0297 |
| | C1 | 6h | 0.3922 | 0.3669 | 0.25 | 0.182(5) | 0.0297 |
| | O1 | 6h | 0.3345 | 0.4711 | 0.25 | 1 | 0.0344 |
| | O2 | 6h | 0.5802 | 0.4610 | 0.25 | 1 | 0.0502 |
| | O3 | 12i | 0.3372 | 0.2566 | 0.0706 | 0.8 | 0.0169 |
| | O4 | 4e | 0 | 0 | 0.1875 | 0.5 | 0.0116 |
| CHA12 | atom | site | x | y | z | occupancy | $U_{iso}$ |
| | Ca1 | 4f | 0.3333 | 0.6667 | −0.0048 | 1 | 0.0235 |
| | Ca2 | 6h | 0.2450 | 0.9901 | 0.25 | 1 | 0.0168 |
| | P1 | 6h | 0.3932 | 0.3674 | 0.25 | 0.780(11) | 0.0133 |
| | C1 | 6h | 0.3932 | 0.3674 | 0.25 | 0.220(11) | 0.0133 |
| | O1 | 6h | 0.3353 | 0.4728 | 0.25 | 1 | 0.0372 |
| | O2 | 6h | 0.5806 | 0.4632 | 0.25 | 1 | 0.0533 |
| | O3 | 12i | 0.3363 | 0.2583 | 0.0675 | 0.8 | 0.0153 |
| | O4 | 4e | 0 | 0 | 0.1760 | 0.5 | 0.0482 |

**Table S4.** Calculated crystallite size and measured average size of CHA nanocrystals.

| Sample | Bragg Angle (°) | hkl | FWHM | Crystallite Size (nm)[a] | Particle Size (nm)[b] | L/D Ratio |
|---|---|---|---|---|---|---|
| CHA1 | 25.98 | (002) | 0.18 | 54.9 | 75.2(L)*22.5(D) | 3.34 |
| CHA2 | 25.96 | (002) | 0.18 | 55.0 | 78.6(L)*21.8(D) | 3.61 |
| CHA3 | 26.02 | (002) | 0.18 | 54.0 | 80.4(L)*24.5(D) | 3.28 |
| CHA4 | 25.86 | (002) | 0.17 | 62.1 | 82.5(L)*25.5(D) | 3.24 |
| CHA5 | 25.92 | (002) | 0.17 | 61.7 | 88.2(L)*27.9(D) | 3.16 |
| CHA6 | 25.94 | (002) | 0.15 | 69.5 | 89.5(L)*30.7(D) | 2.91 |
| CHA7 | 25.96 | (002) | 0.16 | 64.7 | 97.2(L)*32.1(D) | 3.03 |
| CHA8 | 25.90 | (002) | 0.15 | 73.4 | 100.1(L)*32.8(D) | 3.05 |
| CHA9 | 25.96 | (002) | 0.15 | 72.8 | 104.6(L)*34.6(D) | 3.02 |
| CHA10 | 25.91 | (002) | 0.15 | 72.7 | 107.0(L)*32.4(D) | 3.30 |
| CHA11 | 25.96 | (002) | 0.15 | 72.1 | 105.8(L)*34.2(D) | 3.09 |
| CHA12 | 25.91 | (002) | 0.16 | 66.1 | 102.3(L)*34.6(D) | 2.96 |

[a] The crystallite size were calculated by Scherrer's equation. [b] The particle size of the samples were measured and counted by SEM graphs for at least 100 crystal particles.

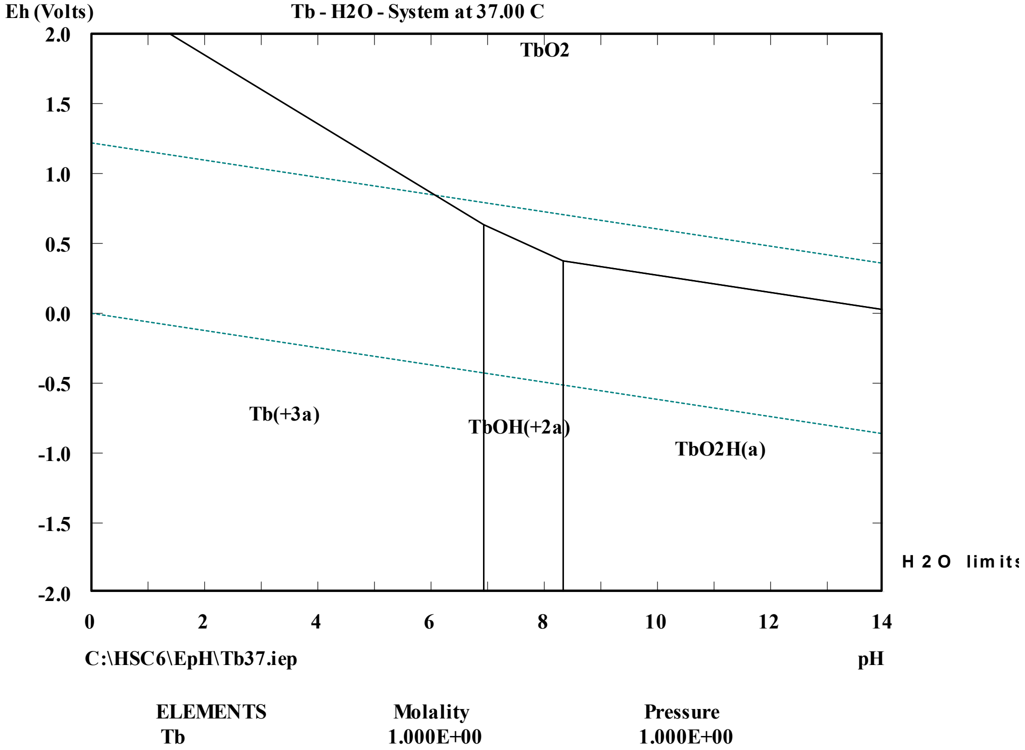


**Figure S12.** Pourbaix diagram of Tb-species in aqueous at 37 °C. For the Pourbaix analysis, Tb-species of Tb, $TbO_2$, $Tb(OH)_3$, $Tb^{4+}$, $Tb^{3+}$, $Tb^{2+}$, TbO(OH), and $TbOH^+$ were accounted with the enthalpy of formation of 0, −217.700, −272.443, −87.524, −159.240, −79.551, −238.170, −205.614 kcal/mol for each species, respectively.

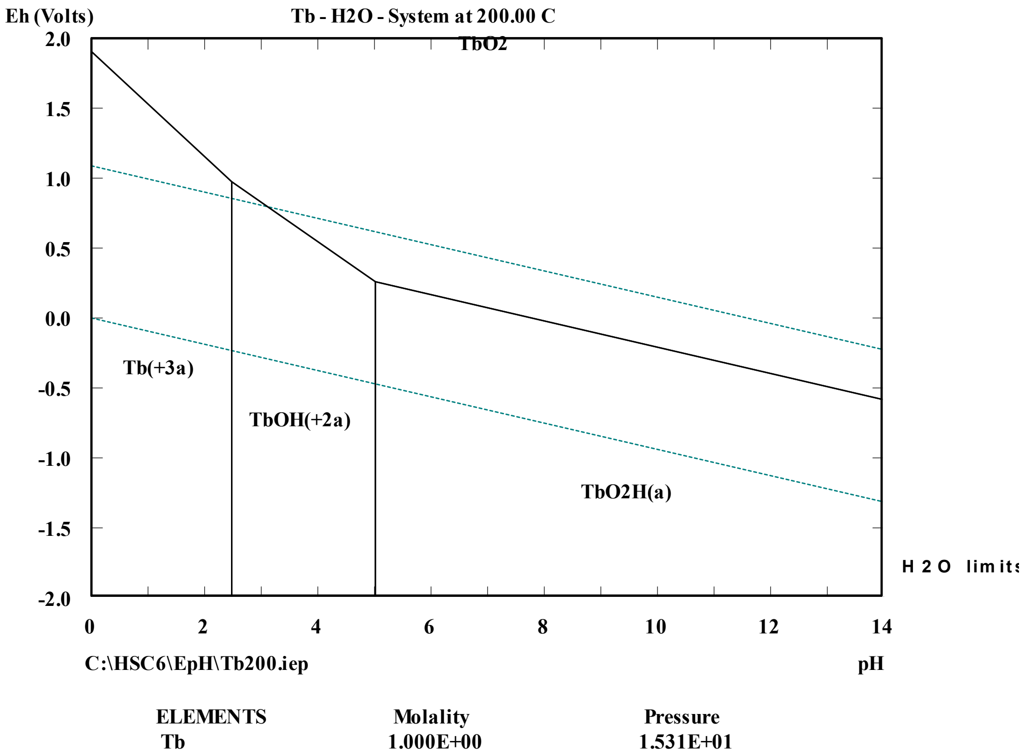


**Figure S13.** Pourbaix diagram of Tb-species in aqueous at 200 °C. For the Pourbaix analysis, Tb-species of Tb, $TbO_2$, $Tb(OH)_3$, $Tb^{4+}$, $Tb^{3+}$, $Tb^{2+}$, TbO(OH), and $TbOH^{+}$ were accounted with the enthalpy of formation of 0, −210.144, −255.025, −77.525, −153.714, −80.876, −226.988, −198.528 kcal/mol for each species, respectively.

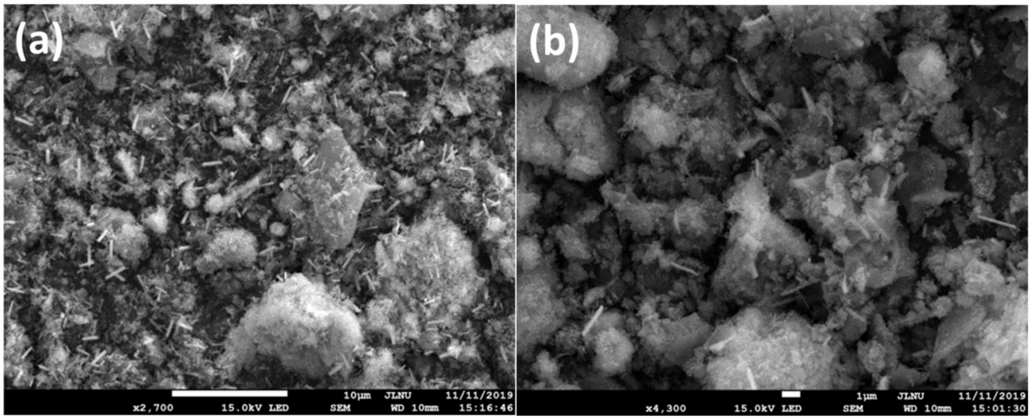


**Figure S14.** SEM of (**a**) HA:5%Tb and (**b**)CHA:5%Tb samples.

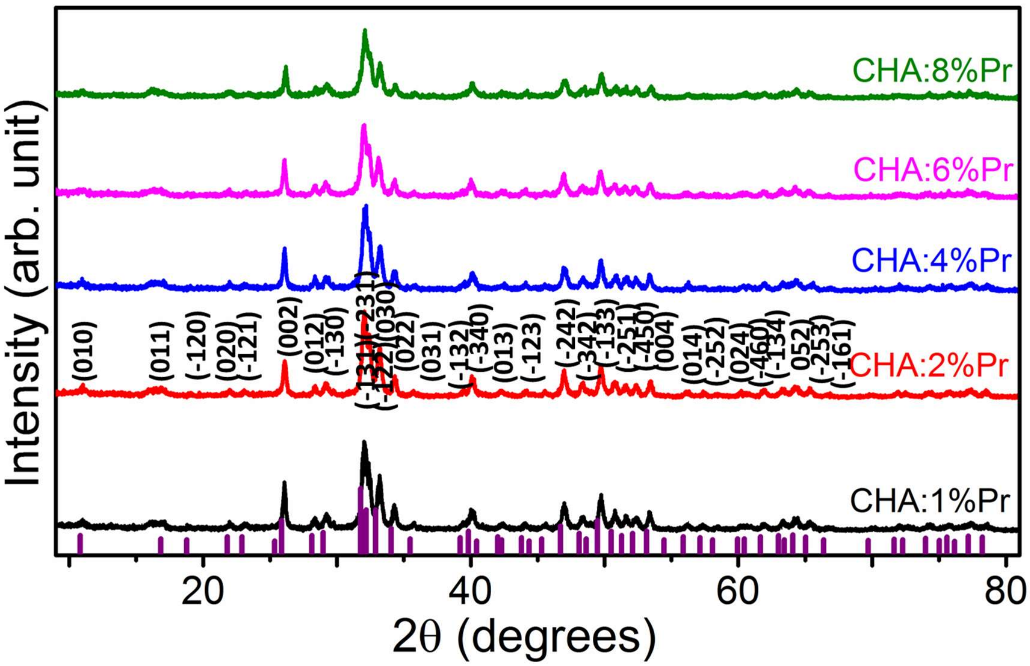


**Figure S15.** PXRD pattern of Pr-doped carbonated hydroxyapatite nanocrystals with increasing doping level from x = 0.01 to 0.08, respectively. Purple bars indicate the theoretical peak positions and intensities of hydroxyapatite with a JCPDS Card No. 09-0432.

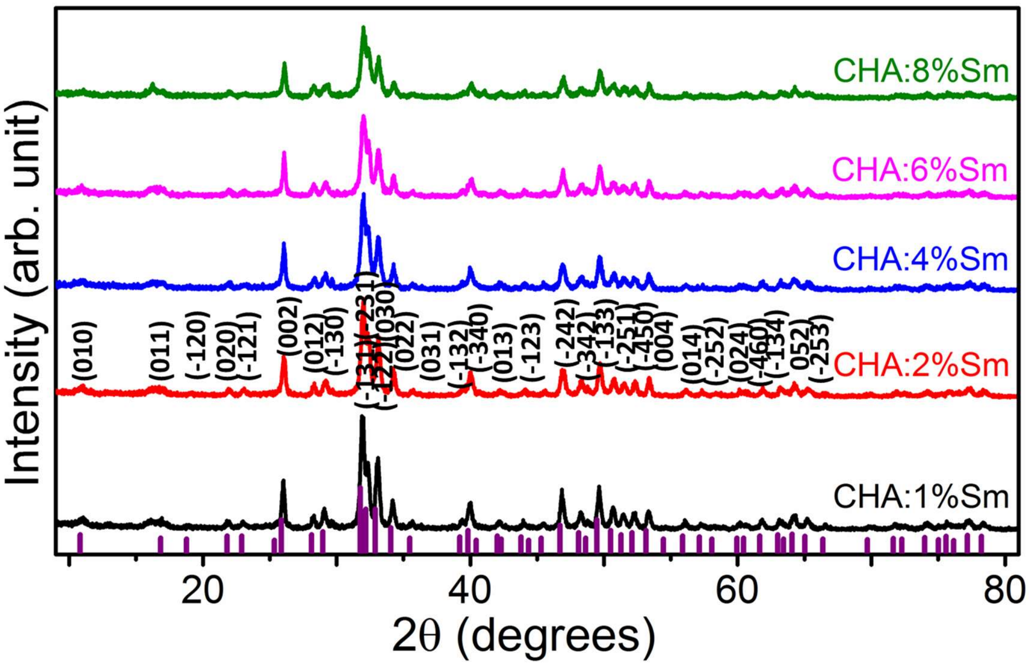


**Figure S16.** PXRD pattern of Sm-doped carbonated hydroxyapatite nanocrystals with increasing doping level from x = 0.01 to 0.08, respectively. Purple bars indicate the theoretical peak positions and intensities of hydroxyapatite with a JCPDS Card No. 09-0432.

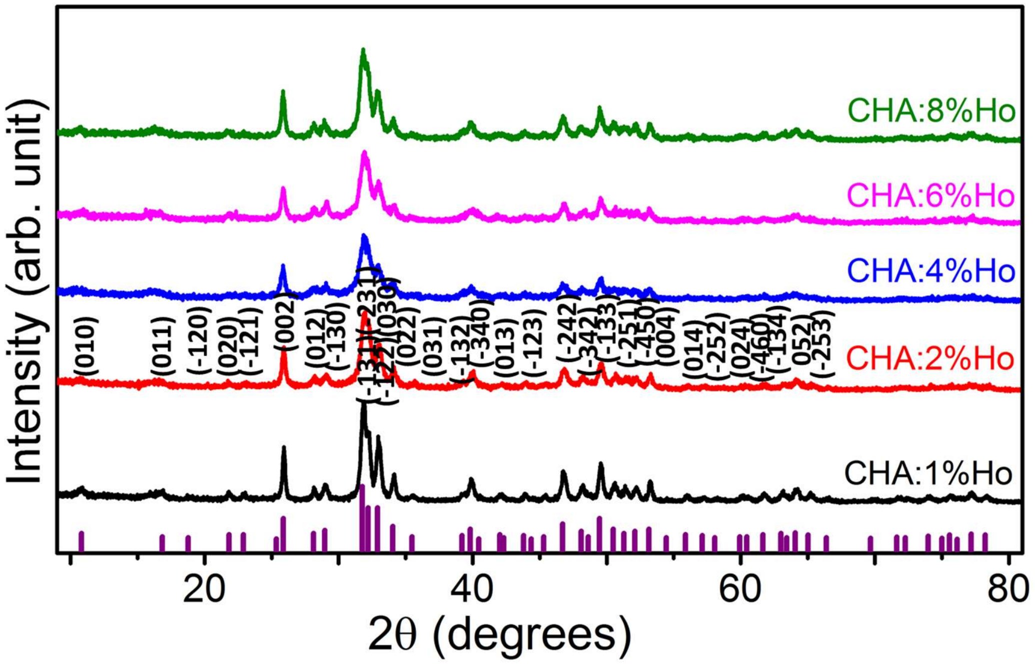


**Figure S17.** PXRD pattern of Ho-doped carbonated hydroxyapatite nanocrystals with increasing doping level from x = 0.01 to 0.08, respectively. Purple bars indicate the theoretical peak positions and intensities of hydroxyapatite with a JCPDS Card No. 09-0432.

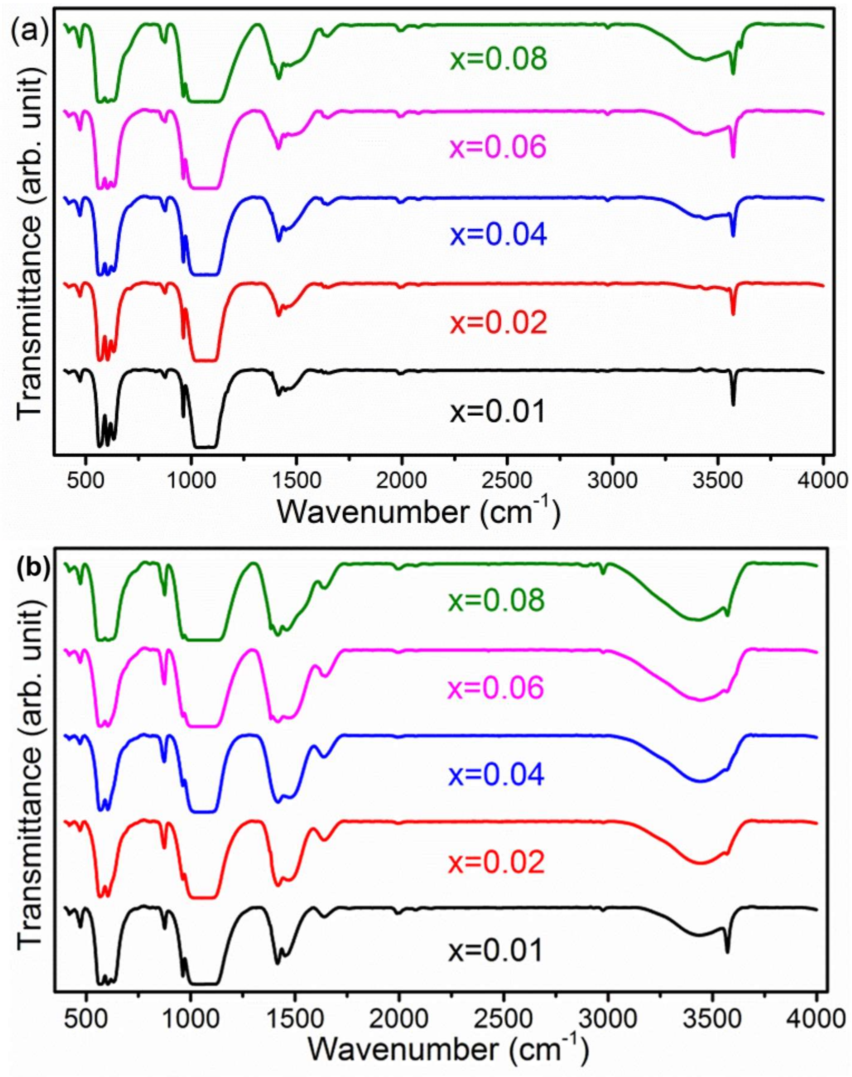


**Figure S18.** FT-IR spectra of various (**a**) $Sm^{3+}$ and (**b**) $Ho^{3+}$ doped hydroxyapatite samples, respectively.

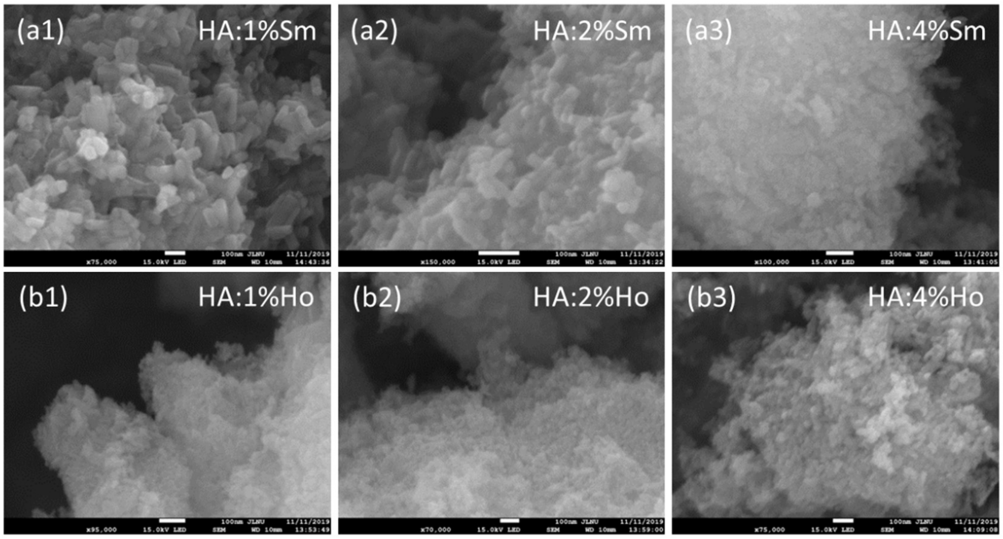


**Figure S19.** SEM graphs of various (**a**) $Sm^{3+}$ and (**b**) $Ho^{3+}$ doped hydroxyapatite samples, respectively.

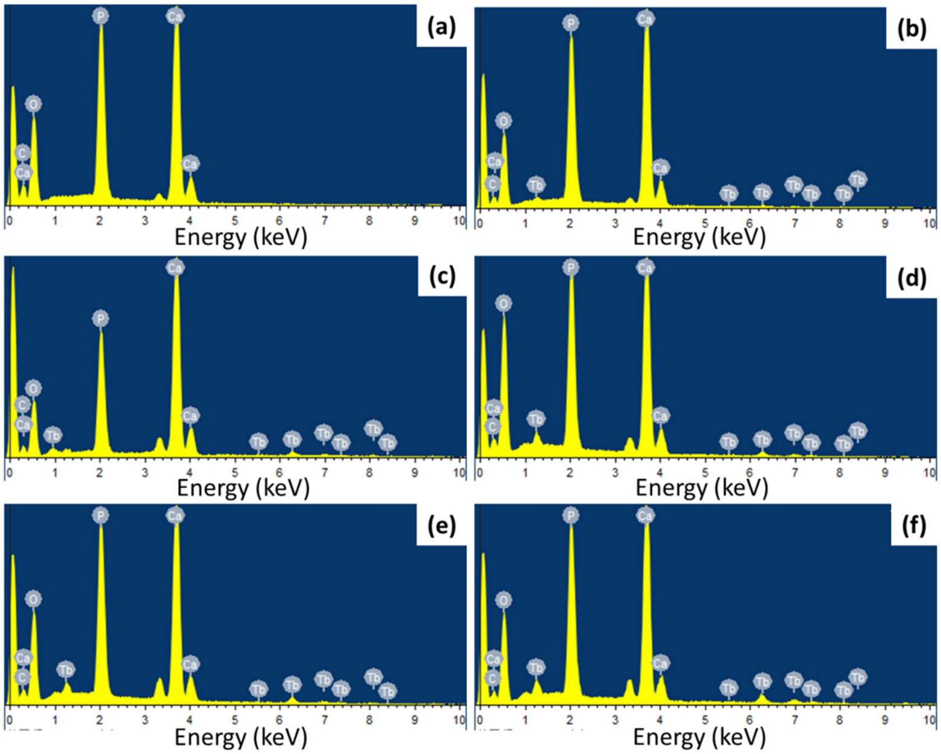


**Figure S20.** EDS spectra of $Tb^{3+}$-doped CHA samples with different $Tb^{3+}$ atomic ratio: (**a**) 0, (**b**) 0.01, (**c**) 0.02, (**d**) 0.03, (**e**) 0.04, and (**f**) 0.05, respectively.

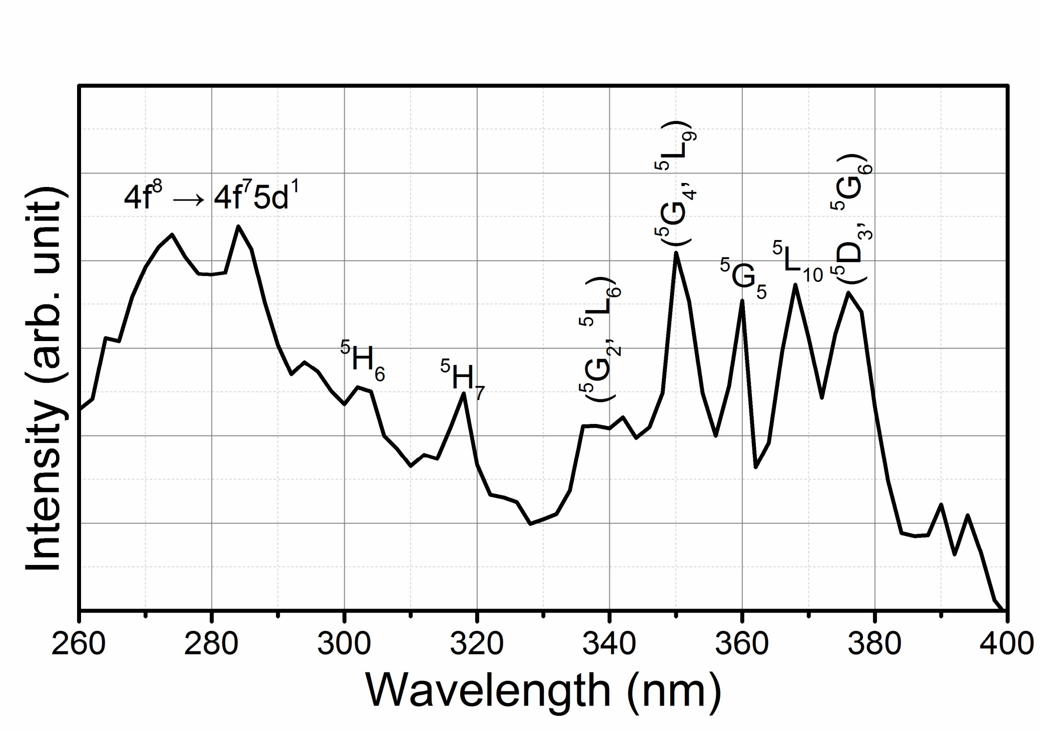


**Figure S21.** Excitation spectra of $Tb^{3+}$ in CHA crystal lattice monitoring the emission at 452 nm.

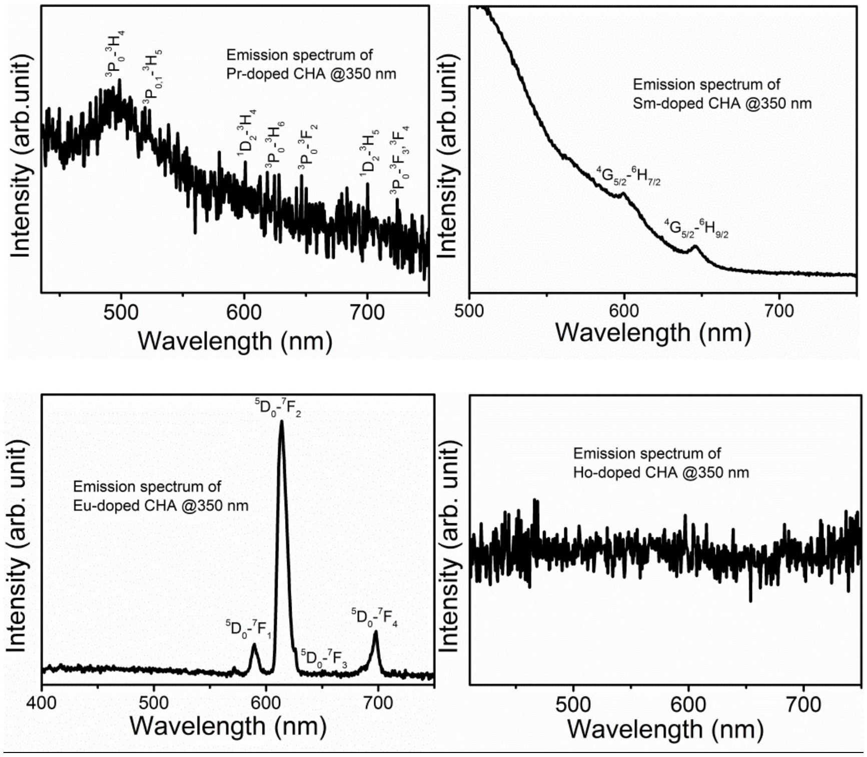


**Figure S22.** Emission spectra of $Pr^{3+}$, $Sm^{3+}$, $Eu^{3+}$, and $Ho^{3+}$ in CHA crystal lattice with the excitation wavelength at 350 nm.

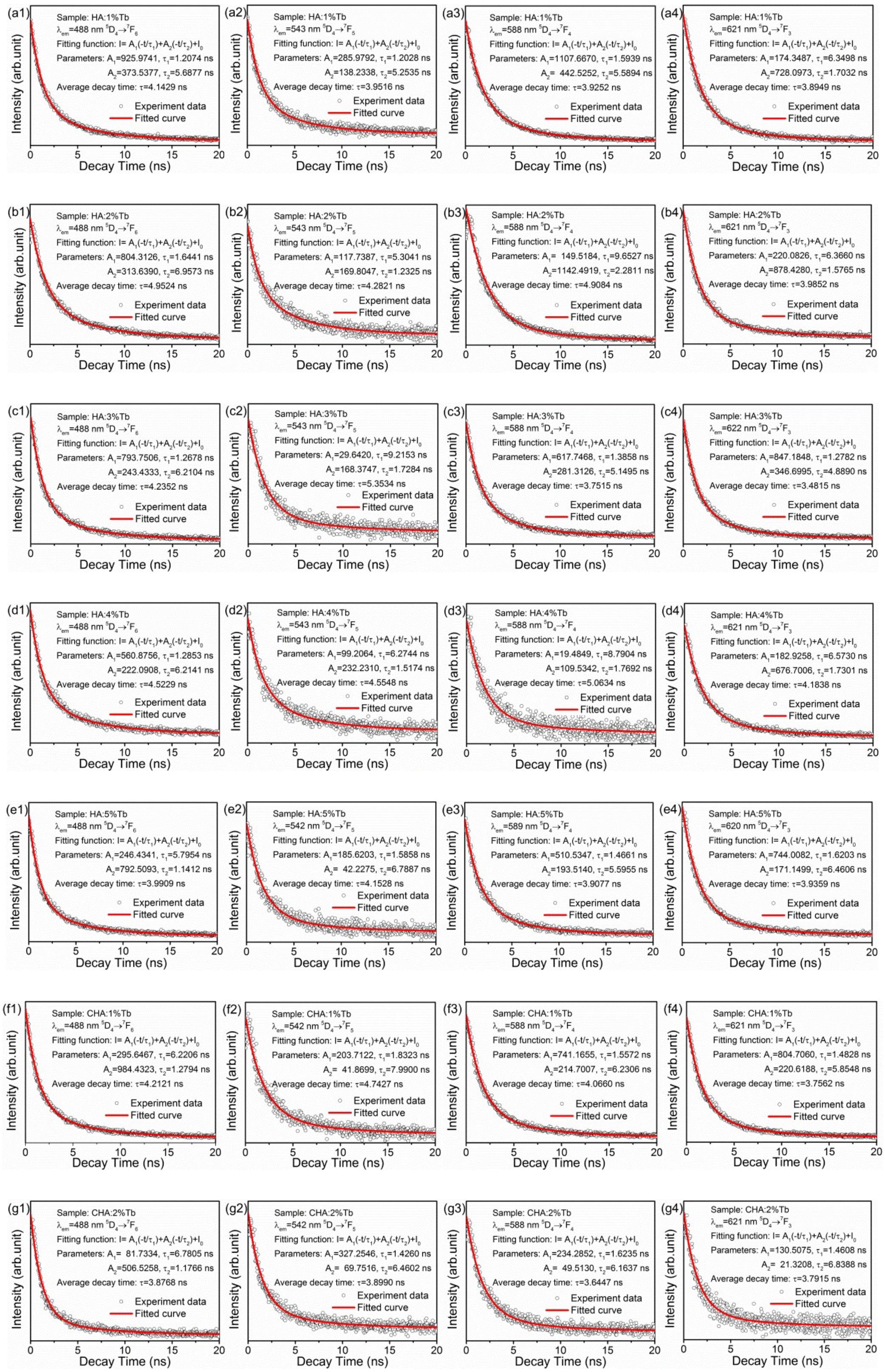
(a1)
Sample: HA:1%Tb
$\lambda_{em}$=488 nm $^5D_4 \to {}^7F_6$
Fitting function: I= $A_1(-t/\tau_1)+A_2(-t/\tau_2)+I_0$
Parameters: $A_1$=925.9741, $\tau_1$=1.2074 ns
$A_2$=373.5377, $\tau_2$=5.6877 ns
Average decay time: $\tau$=4.1429 ns
(a2)
Sample: HA:1%Tb
$\lambda_{em}$=543 nm $^5D_4 \to {}^7F_5$
Fitting function: I= $A_1(-t/\tau_1)+A_2(-t/\tau_2)+I_0$
Parameters: $A_1$=285.9792, $\tau_1$=1.2028 ns
$A_2$=138.2338, $\tau_2$=5.2535 ns
Average decay time: $\tau$=3.9516 ns
(a3)
Sample: HA:1%Tb
$\lambda_{em}$=588 nm $^5D_4 \to {}^7F_4$
Fitting function: I= $A_1(-t/\tau_1)+A_2(-t/\tau_2)+I_0$
Parameters: $A_1$=1107.6670, $\tau_1$=1.5939 ns
$A_2$= 442.5252, $\tau_2$=5.5894 ns
Average decay time: $\tau$=3.9252 ns
(a4)
Sample: HA:1%Tb
$\lambda_{em}$=621 nm $^5D_4 \to {}^7F_3$
Fitting function: I= $A_1(-t/\tau_1)+A_2(-t/\tau_2)+I_0$
Parameters: $A_1$=174.3487, $\tau_1$=6.3498 ns
$A_2$=728.0973, $\tau_2$=1.7032 ns
Average decay time: $\tau$=3.8949 ns
(b1)
Sample: HA:2%Tb
$\lambda_{em}$=488 nm $^5D_4 \to {}^7F_6$
Fitting function: I= $A_1(-t/\tau_1)+A_2(-t/\tau_2)+I_0$
Parameters: $A_1$=804.3126, $\tau_1$=1.6441 ns
$A_2$=313.6390, $\tau_2$=6.9573 ns
Average decay time: $\tau$=4.9524 ns
(b2)
Sample: HA:2%Tb
$\lambda_{em}$=543 nm $^5D_4 \to {}^7F_5$
Fitting function: I= $A_1(-t/\tau_1)+A_2(-t/\tau_2)+I_0$
Parameters: $A_1$=117.7387, $\tau_1$=5.3041 ns
$A_2$=169.8047, $\tau_2$=1.2325 ns
Average decay time: $\tau$=4.2821 ns
(b3)
Sample: HA:2%Tb
$\lambda_{em}$=588 nm $^5D_4 \to {}^7F_4$
Fitting function: I= $A_1(-t/\tau_1)+A_2(-t/\tau_2)+I_0$
Parameters: $A_1$= 149.5184, $\tau_1$=9.6527 ns
$A_2$=1142.4919, $\tau_2$=2.2811 ns
Average decay time: $\tau$=4.9084 ns
(b4)
Sample: HA:2%Tb
$\lambda_{em}$=621 nm $^5D_4 \to {}^7F_3$
Fitting function: I= $A_1(-t/\tau_1)+A_2(-t/\tau_2)+I_0$
Parameters: $A_1$=220.0826, $\tau_1$=6.3660 ns
$A_2$=878.4280, $\tau_2$=1.5765 ns
Average decay time: $\tau$=3.9852 ns
(c1)
Sample: HA:3%Tb
$\lambda_{em}$=488 nm $^5D_4 \to {}^7F_6$
Fitting function: I= $A_1(-t/\tau_1)+A_2(-t/\tau_2)+I_0$
Parameters: $A_1$=793.7506, $\tau_1$=1.2678 ns
$A_2$=243.4333, $\tau_2$=6.2104 ns
Average decay time: $\tau$=4.2352 ns
(c2)
Sample: HA:3%Tb
$\lambda_{em}$=543 nm $^5D_4 \to {}^7F_5$
Fitting function: I= $A_1(-t/\tau_1)+A_2(-t/\tau_2)+I_0$
Parameters: $A_1$=29.6420, $\tau_1$=9.2153 ns
$A_2$=168.3747, $\tau_2$=1.7284 ns
Average decay time: $\tau$=5.3534 ns
(c3)
Sample: HA:3%Tb
$\lambda_{em}$=588 nm $^5D_4 \to {}^7F_4$
Fitting function: I= $A_1(-t/\tau_1)+A_2(-t/\tau_2)+I_0$
Parameters: $A_1$=617.7468, $\tau_1$=1.3858 ns
$A_2$=281.3126, $\tau_2$=5.1495 ns
Average decay time: $\tau$=3.7515 ns
(c4)
Sample: HA:3%Tb
$\lambda_{em}$=622 nm $^5D_4 \to {}^7F_3$
Fitting function: I= $A_1(-t/\tau_1)+A_2(-t/\tau_2)+I_0$
Parameters: $A_1$=847.1848, $\tau_1$=1.2782 ns
$A_2$=346.6995, $\tau_2$=4.8890 ns
Average decay time: $\tau$=3.4815 ns
(d1)
Sample: HA:4%Tb
$\lambda_{em}$=488 nm $^5D_4 \to {}^7F_6$
Fitting function: I= $A_1(-t/\tau_1)+A_2(-t/\tau_2)+I_0$
Parameters: $A_1$=560.8756, $\tau_1$=1.2853 ns
$A_2$=222.0908, $\tau_2$=6.2141 ns
Average decay time: $\tau$=4.5229 ns
(d2)
Sample: HA:4%Tb
$\lambda_{em}$=543 nm $^5D_4 \to {}^7F_5$
Fitting function: I= $A_1(-t/\tau_1)+A_2(-t/\tau_2)+I_0$
Parameters: $A_1$=99.2064, $\tau_1$=6.2744 ns
$A_2$=232.2310, $\tau_2$=1.5174 ns
Average decay time: $\tau$=4.5548 ns
(d3)
Sample: HA:4%Tb
$\lambda_{em}$=588 nm $^5D_4 \to {}^7F_4$
Fitting function: I= $A_1(-t/\tau_1)+A_2(-t/\tau_2)+I_0$
Parameters: $A_1$=19.4849, $\tau_1$=8.7904 ns
$A_2$=109.5342, $\tau_2$=1.7692 ns
Average decay time: $\tau$=5.0634 ns
(d4)
Sample: HA:4%Tb
$\lambda_{em}$=621 nm $^5D_4 \to {}^7F_3$
Fitting function: I= $A_1(-t/\tau_1)+A_2(-t/\tau_2)+I_0$
Parameters: $A_1$=182.9258, $\tau_1$=6.5730 ns
$A_2$=676.7006, $\tau_2$=1.7301 ns
Average decay time: $\tau$=4.1838 ns
(e1)
Sample: HA:5%Tb
$\lambda_{em}$=488 nm $^5D_4 \to {}^7F_6$
Fitting function: I= $A_1(-t/\tau_1)+A_2(-t/\tau_2)+I_0$
Parameters: $A_1$=246.4341, $\tau_1$=5.7954 ns
$A_2$=792.5093, $\tau_2$=1.1412 ns
Average decay time: $\tau$=3.9909 ns
(e2)
Sample: HA:5%Tb
$\lambda_{em}$=542 nm $^5D_4 \to {}^7F_5$
Fitting function: I= $A_1(-t/\tau_1)+A_2(-t/\tau_2)+I_0$
Parameters: $A_1$=185.6203, $\tau_1$=1.5858 ns
$A_2$= 42.2275, $\tau_2$=6.7887 ns
Average decay time: $\tau$=4.1528 ns
(e3)
Sample: HA:5%Tb
$\lambda_{em}$=589 nm $^5D_4 \to {}^7F_4$
Fitting function: I= $A_1(-t/\tau_1)+A_2(-t/\tau_2)+I_0$
Parameters: $A_1$=510.5347, $\tau_1$=1.4661 ns
$A_2$=193.5140, $\tau_2$=5.5955 ns
Average decay time: $\tau$=3.9077 ns
(e4)
Sample: HA:5%Tb
$\lambda_{em}$=620 nm $^5D_4 \to {}^7F_3$
Fitting function: I= $A_1(-t/\tau_1)+A_2(-t/\tau_2)+I_0$
Parameters: $A_1$=744.0082, $\tau_1$=1.6203 ns
$A_2$=171.1499, $\tau_2$=6.4606 ns
Average decay time: $\tau$=3.9359 ns
(f1)
Sample: CHA:1%Tb
$\lambda_{em}$=488 nm $^5D_4 \to {}^7F_6$
Fitting function: I= $A_1(-t/\tau_1)+A_2(-t/\tau_2)+I_0$
Parameters: $A_1$=295.6467, $\tau_1$=6.2206 ns
$A_2$=984.4323, $\tau_2$=1.2794 ns
Average decay time: $\tau$=4.2121 ns
(f2)
Sample: CHA:1%Tb
$\lambda_{em}$=542 nm $^5D_4 \to {}^7F_5$
Fitting function: I= $A_1(-t/\tau_1)+A_2(-t/\tau_2)+I_0$
Parameters: $A_1$=203.7122, $\tau_1$=1.8323 ns
$A_2$= 41.8699, $\tau_2$=7.9900 ns
Average decay time: $\tau$=4.7427 ns
(f3)
Sample: CHA:1%Tb
$\lambda_{em}$=588 nm $^5D_4 \to {}^7F_4$
Fitting function: I= $A_1(-t/\tau_1)+A_2(-t/\tau_2)+I_0$
Parameters: $A_1$=741.1655, $\tau_1$=1.5572 ns
$A_2$=214.7007, $\tau_2$=6.2306 ns
Average decay time: $\tau$=4.0660 ns
(f4)
Sample: CHA:1%Tb
$\lambda_{em}$=621 nm $^5D_4 \to {}^7F_3$
Fitting function: I= $A_1(-t/\tau_1)+A_2(-t/\tau_2)+I_0$
Parameters: $A_1$=804.7060, $\tau_1$=1.4828 ns
$A_2$=220.6188, $\tau_2$=5.8548 ns
Average decay time: $\tau$=3.7562 ns
(g1)
Sample: CHA:2%Tb
$\lambda_{em}$=488 nm $^5D_4 \to {}^7F_6$
Fitting function: I= $A_1(-t/\tau_1)+A_2(-t/\tau_2)+I_0$
Parameters: $A_1$= 81.7334, $\tau_1$=6.7805 ns
$A_2$=506.5258, $\tau_2$=1.1766 ns
Average decay time: $\tau$=3.8768 ns
(g2)
Sample: CHA:2%Tb
$\lambda_{em}$=542 nm $^5D_4 \to {}^7F_5$
Fitting function: I= $A_1(-t/\tau_1)+A_2(-t/\tau_2)+I_0$
Parameters: $A_1$=327.2546, $\tau_1$=1.4260 ns
$A_2$= 69.7516, $\tau_2$=6.4602 ns
Average decay time: $\tau$=3.8990 ns
(g3)
Sample: CHA:2%Tb
$\lambda_{em}$=588 nm $^5D_4 \to {}^7F_4$
Fitting function: I= $A_1(-t/\tau_1)+A_2(-t/\tau_2)+I_0$
Parameters: $A_1$=234.2852, $\tau_1$=1.6235 ns
$A_2$= 49.5130, $\tau_2$=6.1637 ns
Average decay time: $\tau$=3.6447 ns
(g4)
Sample: CHA:2%Tb
$\lambda_{em}$=621 nm $^5D_4 \to {}^7F_3$
Fitting function: I= $A_1(-t/\tau_1)+A_2(-t/\tau_2)+I_0$
Parameters: $A_1$=130.5075, $\tau_1$=1.4608 ns
$A_2$= 21.3208, $\tau_2$=6.8388 ns
Average decay time: $\tau$=3.7915 ns
Experiment data
Fitted curve
Intensity (arb.unit)
Decay Time (ns)
0
5
10
15
20

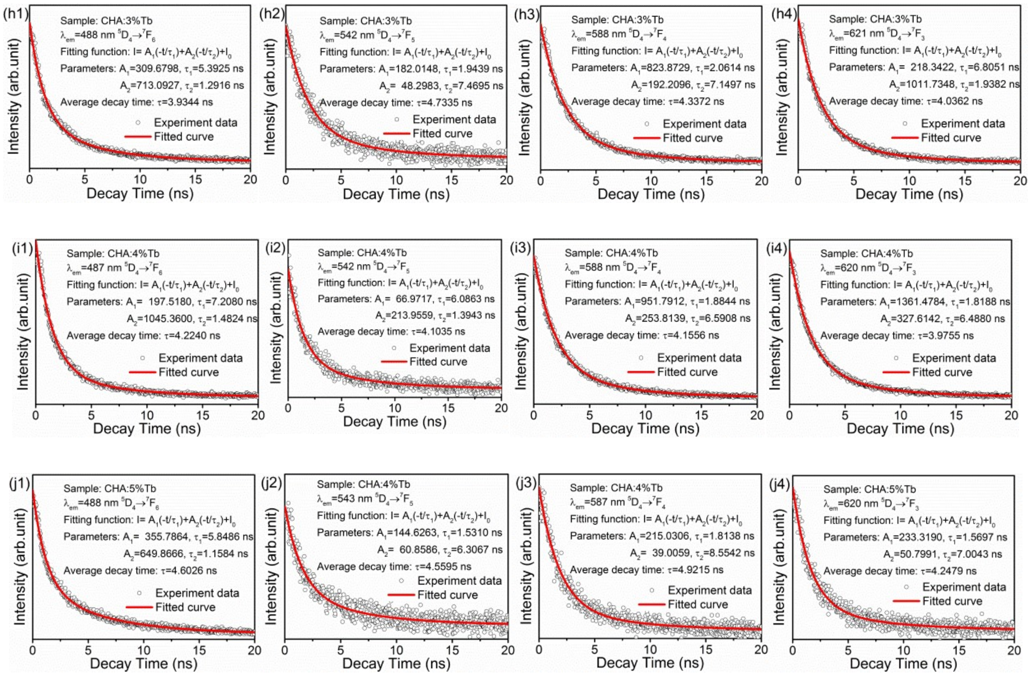


**Figure S23**. Decay times of (**a**) HA:1%Tb, (**b**) HA:2%Tb, (**c**) HA:3%Tb, (**d**) HA:4%Tb, (**e**) HA5%Tb, (**f**) CHA:1%Tb, (**g**) CHA:2%Tb, (**h**) CHA:3%Tb, (**i**) CHA:4%Tb, (**j**) CHA:5%Tb, respectively.

**Table S5.** PL peak position and the fitted results for the decay curve of each apatite samples.

| Material | Peak | Transition | $A_1$ | $\tau_1$ (ns) | $A_2$ | $\tau_2$ (ns) | Average $\tau$ (ns) |
|---|---|---|---|---|---|---|---|
| HA:1%Tb | 488 | $^5D_4$–$^7F_6$ | 925.9741 | 1.2074 | 373.5377 | 5.6877 | 4.1429 |
| | 543 | $^5D_4$–$^7F_5$ | 285.9792 | 1.2028 | 138.2338 | 5.2535 | 3.9516 |
| | 588 | $^5D_4$–$^7F_4$ | 1107.6670 | 1.5939 | 442.5252 | 5.5894 | 3.9252 |
| | 621 | $^5D_4$–$^7F_3$ | 174.3478 | 6.3498 | 728.0973 | 1.7032 | 3.8948 |
| HA:2%Tb | 488 | $^5D_4$–$^7F_6$ | 804.3126 | 1.6440 | 313.6390 | 6.9573 | 4.9524 |
| | 543 | $^5D_4$–$^7F_5$ | 117.7387 | 5.3041 | 169.8047 | 1.2325 | 4.2821 |
| | 588 | $^5D_4$–$^7F_4$ | 149.5184 | 9.6527 | 1142.4920 | 2.2811 | 4.9084 |
| | 621 | $^5D_4$–$^7F_3$ | 220.0826 | 6.3660 | 878.4280 | 1.5765 | 3.9852 |
| HA:3%Tb | 488 | $^5D_4$–$^7F_6$ | 793.7506 | 1.2678 | 243.4333 | 6.2104 | 4.2352 |
| | 543 | $^5D_4$–$^7F_5$ | 29.6420 | 9.2153 | 168.3747 | 1.7284 | 5.3534 |
| | 588 | $^5D_4$–$^7F_4$ | 617.7468 | 1.3858 | 281.3126 | 5.1495 | 3.7515 |
| | 622 | $^5D_4$–$^7F_3$ | 847.1848 | 1.2782 | 346.6995 | 4.8890 | 3.4815 |
| HA:4%Tb | 488 | $^5D_4$–$^7F_6$ | 560.8756 | 1.2853 | 222.0908 | 6.2141 | 4.5229 |
| | 543 | $^5D_4$–$^7F_5$ | 99.2064 | 6.2744 | 232.2310 | 1.5174 | 4.5548 |
| | 588 | $^5D_4$–$^7F_4$ | 19.4849 | 8.7904 | 109.5342 | 1.7692 | 5.0634 |
| | 621 | $^5D_4$–$^7F_3$ | 182.9258 | 6.5730 | 676.7000 | 1.7301 | 4.1838 |
| HA:5%Tb | 488 | $^5D_4$–$^7F_6$ | 246.4341 | 5.7954 | 792.5093 | 1.1412 | 3.9909 |
| | 542 | $^5D_4$–$^7F_5$ | 185.6203 | 1.5858 | 42.2275 | 6.7887 | 4.1528 |
| | 589 | $^5D_4$–$^7F_4$ | 510.5347 | 1.4661 | 193.5140 | 5.5955 | 3.9077 |
| | 620 | $^5D_4$–$^7F_3$ | 744.0080 | 1.6203 | 171.1499 | 6.4606 | 3.9359 |
| CHA:1%Tb | 488 | $^5D_4$–$^7F_6$ | 295.6467 | 6.2206 | 984.4323 | 1.2794 | 4.2121 |
| | 542 | $^5D_4$–$^7F_5$ | 203.7122 | 1.8323 | 41.8699 | 7.9900 | 4.7427 |
| | 588 | $^5D_4$–$^7F_4$ | 741.1655 | 1.5572 | 214.7007 | 6.2306 | 4.0660 |
| | 621 | $^5D_4$–$^7F_3$ | 804.0760 | 1.4828 | 220.6188 | 5.8547 | 3.7562 |
| CHA:2%Tb | 488 | $^5D_4$–$^7F_6$ | 81.7334 | 6.7805 | 506.5258 | 1.1766 | 3.8768 |
| | 542 | $^5D_4$–$^7F_5$ | 327.2546 | 1.4260 | 69.7516 | 6.4602 | 3.8990 |
| | 588 | $^5D_4$–$^7F_4$ | 234.2852 | 1.6235 | 49.5130 | 6.1637 | 3.6447 |
| | 621 | $^5D_4$–$^7F_3$ | 130.5075 | 1.4608 | 21.3208 | 6.8388 | 3.7915 |
| CHA:3%Tb | 488 | $^5D_4$–$^7F_6$ | 309.5798 | 5.3925 | 713.0927 | 1.2916 | 3.9344 |
| | 542 | $^5D_4$–$^7F_5$ | 182.0148 | 1.9439 | 48.2983 | 7.4695 | 4.7335 |
| | 588 | $^5D_4$–$^7F_4$ | 823.8729 | 2.0614 | 192.2096 | 7.1497 | 4.3372 |
| | 621 | $^5D_4$–$^7F_3$ | 218.3422 | 6.8051 | 1011.7350 | 1.9382 | 4.0362 |
| CHA:4%Tb | 487 | $^5D_4$–$^7F_6$ | 197.5180 | 7.2080 | 1045.3600 | 1.4824 | 4.2240 |
| | 542 | $^5D_4$–$^7F_5$ | 66.9717 | 6.0863 | 213.9559 | 1.3943 | 4.1035 |
| | 588 | $^5D_4$–$^7F_4$ | 951.7912 | 1.8844 | 253.8139 | 6.5908 | 4.1556 |
| | 620 | $^5D_4$–$^7F_3$ | 1361.4784 | 1.8188 | 327.6142 | 6.4880 | 3.9755 |
| CHA:5%Tb | 488 | $^5D_4$–$^7F_6$ | 355.7864 | 5.8486 | 649.8666 | 1.1584 | 4.6026 |
| | 543 | $^5D_4$–$^7F_5$ | 144.6263 | 1.5310 | 60.8586 | 6.3067 | 4.5595 |
| | 587 | $^5D_4$–$^7F_4$ | 215.0306 | 1.8138 | 39.0059 | 8.5542 | 4.9215 |

| | | | | | | |
|---|---|---|---|---|---|---|
| | 620 | $^5D_4$–$^7F_3$ | 233.3190 | 1.5697 | 50.7990 | 7.0043 | 4.2477 |
